# Gene Genealogy-Based Mutation Analysis Reveals Emergence of *Aus*, *Tropical japonica*, and *Aromatic* of *Oryza sativa* during the Later Stage of Rice Domestication

**DOI:** 10.3390/genes14071412

**Published:** 2023-07-08

**Authors:** Yingqing Lu

**Affiliations:** 1 State Key Laboratory of Systematic and Evolutionary Botany, Institute of Botany, Chinese Academy of Sciences, 20 Nan Xin Cun, Beijing 100093, China; yqlu@ibcas.ac.cn; Tel.: +86-10-6283-6441; Fax: +86-10-6259-0843; 2 University of Chinese Academy of Sciences, Beijing 100049, China

**Keywords:** *trans* mutations, hybrid origin, mutation density, inner branch, complex phylogeny, GGM analysis, Asian rice

## Abstract

Asian rice (*Oryza sativa* L.) has become a model for understanding gene functions and domestication in recent decades; however, its own diversification is still controversial. Although the division of *indica* and *japonica* and five subgroups (*aus*, *indica* (*sensu stricto*), *japonica* (*sensu stricto*), *tropical japonica*, and *aromatic*) are broadly accepted, how they are phylogenetically related is not transparent. To clarify their relationships, a sample of 121 diverse genes was chosen here from 12 *Oryza* genomes (two parental and ten *O. sativa* (*Os*)) in parallel to allow gene genealogy-based mutation (GGM) analysis. From the sample, 361 *Os* mutations were shared by two or more subgroups (referred to here as *trans* mutations) from 549 mutations identified at 51 *Os* loci. The GGM analysis and related tests indicates that *aus* diverged from *indica* at a time significantly earlier than when *tropical japonica* split from *japonica*. The results also indicate that *aromatic* was selected from hybrid progeny of *aus* and *tropical japonica* and that all five subgroups share a significant number of the early mutations identified previously. The results suggest that *aus*, *tropical japonica*, and *aromatic* emerged sequentially within the most recent 4–5 millennia of rice domestication after the split of *indica* and *japonica*.

## 1. Introduction

Speciation by hybridization is a complex process in nature [1] and can be more difficult to understand when human influence is imposed, as in domesticated plants and animals. Asian rice (*Oryza sativa* L.) was recently shown to come from hybridization between two closely related species, perennial *O. rufipogon* Griff. and annual *O. nivara* Sharma & Shastry, and separate cultivations of the early rice, which took place about 4–5 millennia ago, divided *O*. *sativa* L. into *indica* and *japonica* subspecies [2]. The boundary of the subspecies corresponds to Hsien and Keng in the traditional cultivation of rice in China [3], but variations extend also to aus, tropical japonica, aromatic, and other types of rice that have been long noted in southeast, south and west Asia [3,4]. Collectively, five subgroups have been broadly recognized in cultivated rice, including *indica* (*sensu stricto*), *japonica* (*sensu stricto*), *tropical japonica*, *aus*, and *aromatic* [5]; however, their phylogenetic relationships are still obscure due to contentious evidence for origin of subgroups, as shown below. This is one of the issues to be addressed here, since the system of Asian rice provides a good opportunity for understanding how human activities can shape formation of genetic lineages in crops.

Over the long history of rice domestication, which is estimated to be at least eight millennia [6,7], a great number of cultivars and landraces have developed in each subspecies across Asia [8], with the extant members in each of *indica* (*sensu stricto*) and *japonica* (*sensu stricto*) far outnumbering those in *tropical japonica*, *aus*, and *aromatic*. The five subgroups are supported by genomic variation across a large number of accessions [9,10,11]. Nonetheless, whether or not they share a common early history is unclear, and how *aus* and *aromatic* relate to each other and to other subgroups still remains vague.

Relative to *indica* (*sensu stricto*) and *japonica* (*sensu stricto*) that grow mostly in temperate and subtropical regions of Asia, *tropical japonica*, *aus*, and *aromatic* are mostly cultivated in regions of Asia at a low latitudes. *Tropical japonica*, once called javanica [8], has long been considered an extension of *japonica* in tropical regions, which has few morphological differences from temperate *japonica* but can be differentiated from the latter with molecular markers [12]. In comparison, *aus* was recognized as a clan limited to south and west Asia in an isozyme survey [3]. Relative to *japonica* (*sensu stricto*) and *tropical japonica*, *aus* is closer to *indica* (*sensu stricto*) in not only nucleotide sequences [5] but also in relation to insertion polymorphism of transposable elements [13]. Though frequently less competitive in yield and quality than other rice varieties, *aus* has shown tolerance to low soil phosphate [14,15] or resistance to blast [16] and drought [17]. Since *aus* shows its distinctiveness from both *indica* (*sensu stricto*) and japonica in principal component analysis of genomes [10] and profiling of nucleotide diversity along the chromosomes [18], its status as a branch of *indica* needs more evidence.

Paradoxically, the subgroup *aromatic* has phenotypic similarities to *aus* and *indica* but a SSR similarity to *tropical japonica* [9]. A genomic analysis on 948 landraces identified DNA regions of *japonica, aus,* and *indica* in the circum-Basmati group [19]. Meanwhile, an interrogation of different sets of genomes (nuclear and chloroplastic) suggests a possible origin of *aromatic* rice from interbreeding of *japonica* with a local wild rice in the Indian subcontinent [20]. Genomes of two Basmati varieties show signs of gene flow between the circum-Basmati and circum-aus groups [21]. Still, the origin of *aromatic* has remained speculative.

Rice with aroma is favored by customers worldwide. It contains numerous volatile compounds [22,23], with 2-acetyl-1-pyrroline identified as a major aroma constituent, among others [24,25]. Proline is one of the precursors for acetylpyrroline [26], but the biochemical pathway leading to acetylpyrroline is still elusive. A recessive allele of gene *BADH2* that encodes betaine aldehyde dehydrogenase can partly affect production of acetylpyrroline [27,28]. The roles of other genes (e.g., *BADH1*, [29]) still require more substantiation [30]. Many aromatic cultivars have the same 8 bp deletions in the coding sequence of *BADH2* [27,31,32], but other less frequent mutations also show associations with the trait of fragrance [30,33,34]. In addition to genomic components, the aroma of rice can also be influenced by the growth environment, as shading has a positive impact [35], while grain size and salinity have negative impacts on the intensity of aroma of rice [36,37].

With more quality genomes released publicly, it is possible to carry out a genome-wide analysis on all recognized subgroups of Asian rice using the gene genealogy-based mutation (GGM) analysis lately reported [2] and relevant information from various disciplines (e.g., genomes of wild relatives, life-history traits, geographic distributions, gene functions, fossils, records of introgression, etc.). The goal of this study is to use fixed mutations specific to Asian rice to reconstruct phylogenetic relationships among subgroups during domestication. The key differences of this method from a phylogenomic approach are the classification of SNPs temporally and spatially via gene genealogies and the selection of a subset of SNPs and indels that are most relevant to phylogeny to build a reconstruction with minimum restrictions. Instead of evaluating the total biodiversity of Asian rice, the method focuses more on genes of known structures than of unknown structures in order to reduce errors arising from misidentification of mutations in different regions. It assumes that the bias of gene sampling is negligible in the reconstruction of a phylogeny since the phylogenetic relationships have the same impacts on all genes. As the accuracy and precision of mutation identification are vital to the GGM analysis, sampling of genomes was performed in representative lines of the five subgroups which have been well sequenced to allow reliable identifications of mutations. Here, an allelic perspective (instead of SNP or haplotype) is adopted since the known ancestors of Asian rice permit direct identifications of mutations at the genic level, and gene genealogies can be easily built. The footprints left in the genomes will be examined in situ to infer historical events. It is shown that an extensive analysis of mutational distributions on gene genealogies including all five subgroups can provide a clear interpretation for the origin of *aromatic* rice while clarifying phylogenetic relationships among the current cultivars of *aus*, *tropical japonica*, *aromatic*, *indica* (*sensu stricto*) and *japonica* (*sensu stricto*). A more complete image of rice domestication outside the Yangtze River begins to emerge, showing how the crop has been changed over millennia of ceaseless cultivation by human populations.

## 2. Materials and Methods

### 2.1. Study Samples

A total of 12 nuclear genomes (Table S1) were consulted for the following analysis, which were chosen based on the standards of a high coverage (>100×, except N22 of *aus* (65×)) and quality methods of sequencing and assembly. Genomes of *indica* (*sensu stricto*) were sampled from three varieties (9311, Shuhui498, and Minghui63) to confirm mutations in this lineage, as all three genomes were sequenced only by the PacBio technique (though at a far deeper coverage than those of other genomes) that has a higher error rate than the second-generation techniques. The genomes, representing five subgroups of *O*. *sativa* (*Os*) and two progenitors, *O. rufipogon* (*Or*) and *O. nivara* (*On*), were downloaded from the NCBI data base (www.ncbi.nlm.nih.org) prior to 2 May 2021. The genes sampled for gene genealogies were mainly from the list of Lu et al. [2], as well as some additional genes, which were selected regardless of their location, function, or size.

### 2.2. Gene Genealogy-Based Reconstruction of Intra-Specific Phylogeny

#### 2.2.1. Classification of Mutations Based on Gene Genealogies

Since a conventional SNP- or haplotype-based phylogenetic (or phylogenomic) reconstruction typically requires no selection or recombination, it is inappropriate for Asian rice, as the assumptions are clearly violated given its hybrid origin. With no such restrictions, a gene genealogy-based mutation analysis [2] was adopted here in a phylogeny reconstruction. It builds on the known relationship of Asian rice with its parental species (*O*. *nivara* and *O*. *rufipogon*). Both 5′ and the coding regions were used but treated separately to show regional patterns of genes. Mutations (including both indel and substitution) in *O*. *sativa* were identified by aligning orthologous sequences across 12 genomes including the parental species (Figure S1). All the *Os* mutations were classified as *trans*-subgroup (*trans*) or subgroup-specific. This step is essential in our analysis, since *trans* mutations occurred earlier and entirely during traditional breeding. They are, therefore, closely associated with the past history of rice domestication. Another significant feature of *trans* mutations is that they are represented at least twice in an alignment, which suggest that they tend to be mutations of historically high frequencies. Fixed mutations are preferred over transient ones, because they contribute more steadily to the divergence between subgroups. The *trans* mutations across all five subgroups are presented in a table format (Figure S1), with the sites polymorphic between the ancestral lineages but involving no new mutations in *O*. *sativa* omitted to simplify the presentation. This step excluded many SNPs present between the parental species. When showing the associations of mutations among lineages, however, the figures typically include all polymorphic sites, following the previous format of gene genealogy [2], to give the context for new changes. Additional details on the counting of *trans* mutations are given in Figure S1.

#### 2.2.2. Gene Genealogies Sampled for Phylogenetic Reconstruction

A gene genealogy is not to be confused with a phylogeny simply by their resemblance in topology. Many samples can be taken from relevant genomes to build gene genealogies, but the phylogeny to be reconstructed is the same. While the former is a ‘gene tree’ and the latter a ‘tree of organisms’, the GGM method uses a sample of ‘gene trees’ to infer a phylogeny. It does so by stratifying *Os* mutations on gene genealogies (or its simplified table format) to separate mutations in time/space. The method is particularly suitable for a recently diverged group of taxa since their genomes can be easily sampled to allow reliable identifications of mutations without worrying about repeated substitutions across sites. The organized data enable various analyses, statistical tests, and inferences on a phylogeny. It is specified here that on a phylogeny, all branches are divided into terminal branches and inner branches. Terminal branches directly connect subgroups and are mostly influenced by subgroup-specific mutations. An inner branch here refers to either an internal branch (a branch between two adjacent nodes (branching points)) or a branch between any connecting nodes on a phylogeny, which in either case is mainly defined by *trans* mutations, according to the GGM method. Since the minimum number of gene genealogies needed for a phylogenetic reconstruction varies with each biological system, it is not atypical to sample hundreds of loci for gene genealogies.

#### 2.2.3. Inference of Phylogeny from Distributions of Mutations among Subgroup Combinations

To start the analysis, all the genes sampled were screened for distributions of *trans* mutations. These mutations were further classified into bins of permuted subgroup combinations (two-subgroup combinations, three-subgroup combinations, etc.). Phylogenetically informative combinations are expected to gather more *trans* mutations than random combinations. The bins having more mutations or loci than those compatible to randomness are the top candidates for inference of phylogenetic associations. These candidates can be further tested, if needed, with additional gene genealogies, or examined for succession of their emergences via comparisons of inner branches prior to the divergence of associated subgroups. The relative lengths between inner branches of a phylogeny can be compared via distributions of *trans* mutations by a measure called mutation density (*m_d_*), which is defined here as number of *trans* mutations per nucleotide of a gene region (e.g., 5′, or coding) per branch (period of comparison). It is analogous to nucleotide divergence (π) but includes both indels and substitutions from 5′ or coding regions as an indel mutation and a substitution mutation are treated non-differentially. The mean of mutation densities across the sampled gene genealogies for a given branch is used here to infer the branch length of a phylogeny since it measures the relative divergence of the relevant genomes over the period. Factors that are expected to influence the length of a branch on a phylogeny can be modeled as below.

Let a period associated with an inner branch be T on a phylogeny, the mutation rate per individual be *v* per generation or *u* per nucleotide site per generation, and the selection intensity be *s*. Then, the expected number of fixed mutations (*m*) on the branch is 2*vN_e_*PT under a deterministic model [2] that assumes a large population and steady environment [38], where *N_e_* is the effective population size, T is the unit of the total generations associated with the branch, and P is the probability for a mutation to be fixed in Asian rice. Here we consider the number of fixed mutations on an inner branch (*m_i_*) with a relatively small sample size of genes, and variation in *v* due to gene size can be ignored by replacing *v* with *u*. Let L be the nucleotide number of a gene region under consideration. Then, the expected number of mutations on an inner branch becomes 2L*uN_e_*PT. From the definition above, *m_d_* = *m_i_*/L = 2*uN_e_*PT.

Let N be the size of a breeding population; unlike sizes of cultivated populations, it can be more or less steady under traditional breeding of rice. *N_e_* is half of N [39]. The observed mutations (m_d_) on an inner branch can be approximated by *u*NPT, following the argument above. Since P is primarily a function of *s*, and *s* is relatively steady over time under the traditional cultivation, P can be approximated by a constant in Asian rice. The observed mutations (m_d_) on an inner branch can therefore be further approximated simply by T in an arbitrary unit of *u*NP (assumed collectively a constant during the domestication of rice). Under these conditions, an observed m_d_, which is proportional to T, can be compared between inner branches of the same phylogeny via a statistical test. The results of the test allow us to infer the order of emergence of the inner branches. For instance, under the null hypothesis of no difference in T between branches A and B, the observed values of m_d_ for A and B, averaged over sampled genes, can be compared by Student’s *t*-test or other appropriate tests. If B > A, given that a larger m_d_ is expected to be associated with a longer time period before a given branching point, rejection of the hypothesis can lead to an inference of later divergence of the branch B. Here, *t*-tests of m_d_ were carried out between the branch leading to *aus* and *indica* (*sensu stricto*) (A) and one to *tropical japonica* and *japonica* (*sensu stricto*) (B) on the phylogeny connecting the subgroups of *O*. *sativa* to its progenitors.

In sampling of genes, those known to be under the influence of pleiotropy or epistasis should be used with caution as mutation patterns may vary due to their impacts on the number and independence of mutations. When the information is not available, a large sample size of genes may help as pleiotropy tends to be restricted rather than universal in genomes [40].

## 3. Results

### 3.1. All Five Subgroups Share the Same Early Mutations

A total of 121 genes were surveyed from the sample of 12 nuclear genomes of Asian rice and its wild relatives (Table S1), which yielded 42 phylogenetically informative loci in *O*. *sativa* from those previously listed (Table S1 of Lu et al. [2]) and 9 more phylogenetically informative loci from 20 additional genomic regions (Table S2) due to the presence of *trans* mutations at the loci. Since genes of various functions and sizes, both known and unknown, were sampled across 12 chromosomes of *O*. *sativa* (Table S3), the patterns observed can be taken as a genome-wide phenomenon.

The 51 loci of *O*. *sativa* (Table 1) had 549 identified mutations, 361 of which were *trans* mutations shared by at least two subgroups (Figure S1a–l). Significantly, 116 of the *trans* mutations were present in subgroups of at least *indica* (*sensu stricto*) and *japonica* (*sensu stricto*) at 29 loci, including 42 at 18 loci present in all five subgroups (Table 1). The proportions of the mutations commonly shared among the five subgroups (35% for sampled loci and 12% of all *trans* mutations) cannot be explained by random sorting of *trans* mutations or other chance events. For instance, random sorting of the sampled 361 mutations under a uniform distribution could only explain, on average, about 14 mutations at two loci for mutations in the class of five subgroups, among 26 possible combinations. Yet, the observed pattern of *trans* mutations in the five subgroups is in line with the early history of rice domestication previously identified [2]. Many of the 19 loci (e.g., *SH4*, *Rc*, *RAE2*, *TCP19*, *GL3.2*, *DFR*, and *DAHPS2*) were previously tested under positive selection [2]. The 42 *trans* mutations largely came from fixed early mutations that are expected to pass down constantly to later generations.

### 3.2. Subgroup Aus Differentiated in Indica and Tropical Japonica in Japonica

**Figure 1:**
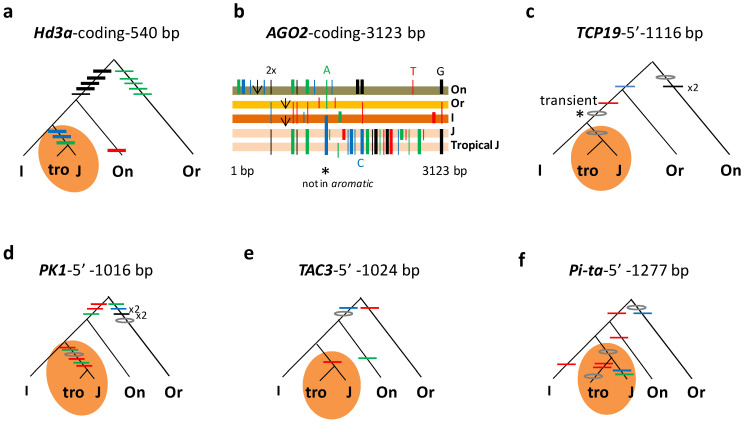
Gene genealogies showing the association of *tropical japonica* (tro) with *japonica* (J, *sensu stricto*). Gene genealogies are shown for six loci, with relevant mutations indicated in the highlighted circle (orange), following the format of Lu et al. (2022). Nonsynonymous substitutions are in thick bars (black for nucleotide G, green for A, red for T, and blue for C), and indels in flattened circles or arrows. Repeated changes are indicated by the number after x. An early mutation is shown in *. An ortholog from indica (I, *sensu lato*) is included, along with those from ancestral species (*O*. *nivara* (On) and *O*. *rufipogon* (Or)) as outgroups. Genealogies are based on coding sequences of *Hd3a* (**a**) and *AGO2* (**b**), or 5′ regions of *TCP19* (**c**), *PK1* (**d**), *TAC3* (**e**), and *Pi-ta* (**f**).

**Figure 2:**
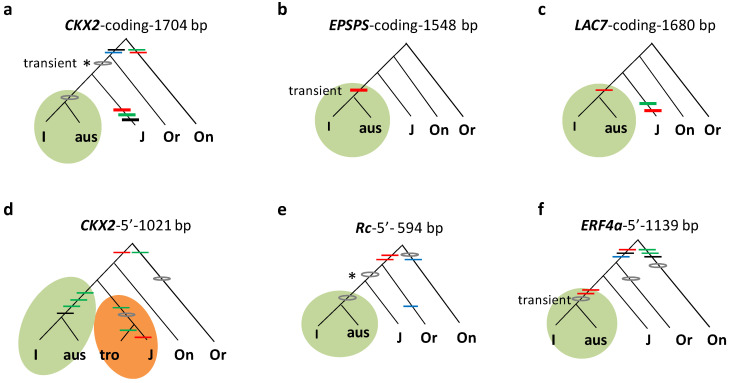
Specific mutations shared between subgroups *aus* and *indica* (I, *sensu stricto*). Gene genealogies are given at five loci (**a**–**f**). The format follows Figure 1. Genealogies are based on the coding sequences of *CKX2* (**a**), *EPSPS* (**b**), and *LAC7* (**c**) or 5′ regions in *CKX2* (**d**), *Rc* (**e**), and *ERF4a* (**f**). An early mutation is shown in *. The colored branches highlight specific *trans* mutations within subspecies (*indica* in green, *japonica* in orange).

Of the 361 *trans* mutations above, 126 are specific to two subgroups. A majority (59%) of the 126 *trans* mutations were concentrated in two combinations, *japonica* (*sensu stricto*)–*tropical japonica* and *indica* (*sensu stricto*)–*aus* (Table 1 and Figure S1). A total of 49 *trans* mutations from 11 loci at 5′ and/or coding regions were specifically shared between *japonica* (*sensu stricto*) and *tropical japonica*, as shown at six representative loci (Figure 1). This suggests that *tropical japonica* and *japonica* (*sensu stricto*) had a common history prior to their differentiation. Meanwhile, 26 *trans* mutations at 13 loci specifically occurred in *indica* (*sensu stricto*) and *aus*, which also indicates their joint history before the split of *aus* and *indica* (*sensu stricto*). Five representative loci exhibit how these mutations unite the subgroups within the *indica* subspecies (Figure 2). In both cases, the extensive sharing of specific mutations have little to do with random events or natural introgressions since natural introgression is rare in rice and outcrossing rates are typically less than 1% in a natural field [41]. Phylogenetic associations are the major reason for patterns of enriched *trans* mutations in the specific combinations of subgroups.

Besides the two combinations above, six of the ten possible combinations of two subgroups have fewer than nine *trans* mutations (Figure S1). Specifically, only one gene shows one *trans* mutation at *GS5* between *aromatic* and *indica* (*sensu stricto*), one *trans* mutations at *Hd1* between *aus* and *tropical japonica*, three *trans* mutations at an unknown locus (tentatively *ACS3*) between *aromatic* and *japonica* (*sensu stricto*), and four *trans* mutations at *DFR* between *aus* and *japonica* (*sensu stricto*); two genes show three *trans* mutations at *GL3.2* and *SPL13* between *tropical japonica* and *indica* (*sensu stricto*) and eight *trans* mutations in *ACS3* and *Hd1* between *japonica* (*sensu stricto*) and *indica* (*sensu stricto*). These sporadic distributions are more or less compatible with random events and provide little information for phylogeny. Two combinations, *aromatic*–*aus* and *aromatic*–*tropical japonica*, however, show more mutations at more loci and are examined below.

### 3.3. Subgroup Aromatic Was Derived from Hybrid Progeny between Aus and Tropical Japonica

**Figure 3:**
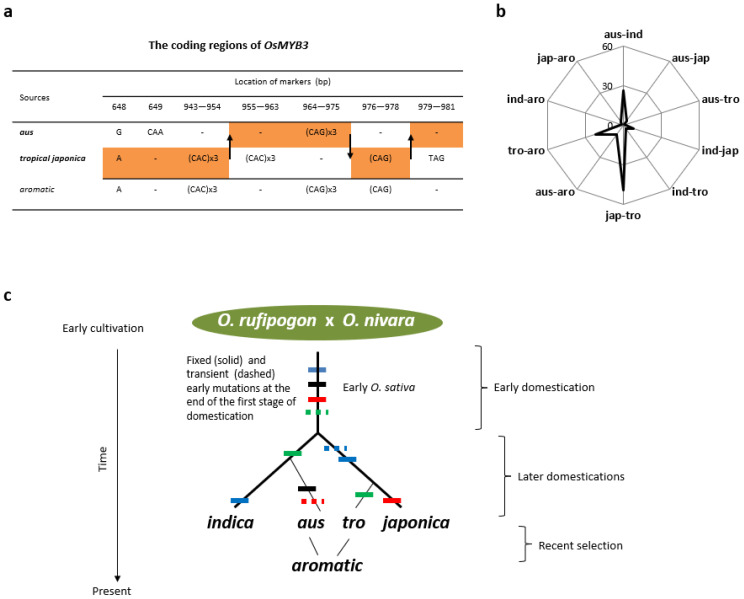
Events leading to phylogenetic relationships among subgroups of Asian rice. (**a**) Recombination events detected at *MYB3*. The sequences from three representative genomes were aligned over 1053 sites of the coding regions. The arrows indicate three likely events of crossing-over between chromatids of *aus* and *tropical japonica* (in bold) in early *aromatic*, which led to the *aromatic* allele via reconnecting the gene regions in yellow. Nucleotide positions are labeled partly around the recombination events. (**b**) A radar graph on the distribution of 126 *trans* mutations among ten classes of two-subgroup pairings. The five subgroups are ind (*indica sensu stricto*), jap (*japonica sensu stricto*), tro (*tropical japonica*), aus (*aus*), and aro (*aromatic*). (**c**) The phylogeny of Asian rice. The subgroups (*sensu* Garris et al., 2005) are in italic and bold. Mutations are featured by the colored bars in each lineage (dashed for transient and solid for fixed mutations). Major stages of domestication (after parenthesis) follow the evidence presented here for the later domestication and in Lu et al., 2022 [2] for the early domestication. The branches leading to *aus*, *tropical japonica*, and *aromatic* are not proportional.

The top two subgroups that *aromatic* shared *trans* mutations with were *tropical japonica* and *aus*. Table 2 lists 13 loci in *aromatic* that contain 36 mutations specifically shared with either *aus* (14 at 5 loci) or *tropical japonica* (22 at 8 loci). Given the origins of *tropical japonica* and *aus* in two subspecies, their relationships with *aromatic* are more consistent with gene flow than with sharing of a common ancestor as it is difficult for *aromatic* to derive from two different lineages unless it is a hybrid. There are at least two cases from the sampled genomes that clearly indicate gene flows from *aus* and *tropical japonica* to *aromatic* and not *vice versa*. One case is the coding region of *MYB3* on chromosome 3, which displays an *aromatic*-specific sequence that appears to be a recombinant of *aus* and *tropical japonica* alleles after three crossing-over events identified at the regions (Figure 3a). Another case is the coding region of *Hd1* on chromosome 6, which shows that the *aromatic* allele came from two recombination events between *aus* and *tropical japonica*, encoding a significantly altered Hd1 (Figure S2). The scenario of gene flows from *aus* and *tropical japonica* to *aromatic* is also consistent with patterns of loci having new mutations across terminal branches. It is expected that the earlier the emergence time of a subgroup, the more loci are likely under human selection when other conditions are similar. In comparison with the number of loci having mutations specific to *tropical japonica* (14) or *aus* (21), *aromatic* has nine loci that contain at least one *aromatic*-specific mutation at either 5′ and/or coding regions (Figure S1). This lowest number among the three subgroups indicates that *aromatic* is a relatively young subgroup, which is consistent with its emergence as hybrid progeny of *aus* and *tropical japonica*.

To collect more evidence of subgroup-specific selection, *BADH2* in *aromatic* was examined and only one deletion in its 5′ region was seen; by contrast, the gene in *aus* contains multiple mutations (Figure S3). In addition to *MYB3* shown above, *aromatic*-specific alleles were also observed at *GL3.2* (Figure S1c), *TCP19* (Figure S1f), and *MYC2* (Figure S1j).

### 3.4. Subgroup Aus Differentiated from Indica Earlier than Tropical Japonica from Japonica

To understand why *trans* mutations are more abundant between *tropical japonica* and *japonica* (*sensu stricto*) than between *aus* and *indica* (*sensu stricto*), the likelihood that the two bifurcating (selective) events that caused the split of the subgroups happened at different times of rice domestication was tested. Given that *aromatic* inherited specific mutations from *aus* and *tropical japonica*, the *trans* mutations it shared with *aus* or *tropical japonica* were also included in the tests. These mutations came from the same period before the emergence of *aus* and *indica* (*sensu stricto*) or *tropical japonica* and *japonica* (*sensu stricto*) and happened to be sorted into *aromatic* during the hybridization detected above. This led to a total of 156 *trans* mutations (37 in coding and 119 in 5′ regions) from 33 loci at 5′ and/or coding regions allocated to the period immediately before the split of *tropical japonica* and *japonica* (*sensu stricto*). Meanwhile, 29 *trans* mutations (12 in coding and 17 in 5′ regions) from 16 loci at 5′ and/or coding regions are specific to the branch leading to the split of *indica* (*sensu stricto*) and *aus* (Table 1). The average mutation densities on the internal branch leading to the split of *aus* and *indica* (*sensu stricto*) were 0.0017 (s.e. 0.0004, n = 10) for 5′ regions and 0.0010 (s.e. 0.0001, n = 10) for coding regions. Those on the branch prior to *tropical japonica* and *japonica* (*sensu stricto*) were 0.0035 (s.e. 0.0007, n = 32) and 0.0019 (s.e. 0.0004, n = 16), respectively (Table 1). The comparisons of mutation densities were made over the longer time of the two internal branches, with the assumption that mutations are similarly distributed over the time on the branches. Under the null hypothesis of no difference in mutation density between branches, results of one-sided *t*-tests indicate that the internal branch before *tropical japonica* (and *japonica* (*sensu stricto*)) was significantly longer (about two times) than the one preceding *aus* (and *indica* (*sensu stricto*)), and the pattern held at both 5′ (*p* = 0.010, n_aus_ = 10, n_tro_ = 32) and coding regions (*p* = 0.017, n_aus_ = 10, n_tro_ = 16). In other words, *aus* branched off significantly earlier in *indica* to form its own lineage, whereas *tropical japonica* separated from *japonica* (*sensu stricto*) significantly later.

Additional evidence is in line with the statistical result above. First, alleles in *aus* inherited more *O*. *nivara* (or sometimes *O*. *rufipogon*) type at various loci (*MYB3*, *Hd6*, *EPSPS*, *SPL16*, *PGI*, *ACS3*, and *WG7*) than alleles of other subgroups (Figure S1). This may stem from its early divergence in *indica* since *indica* inherited more alleles from *O*. *nivara* than from *O*. *rufipogon* during its initial selection [2]. Secondly, when transient mutations segregated with ancestral nucleotides, *aus* frequently kept the ancestral ones, as shown by several 5′ mutations at *Hd6* and *GL3.2* (Figure S1c) and three transient mutations (two in 5′ and one in the coding regions) at the *Rc* locus (Figure S1g). Obviously, early divergence of *aus* could directly lead to its low sharing of mutations with *indica* (*sensu stricto*).

### 3.5. Phylogeny Reconstructed from Collective Evidence

Together, four pairs of subgroups (*tropical japonica–japonica* (*sensu stricto*), *aus*–*indica* (*sensu stricto*), *aromatic–tropical japonica*, and *aromatic–aus*) accounted for 84% (106 out of 126) of the *trans* mutations specific to two subgroups (Figure 3b). The distribution gives the basic structure of the phylogeny of Asian rice, along with the analysis above showing that the subgroups *aus*, *tropical japonica*, and *aromatic* emerged sequentially during domestication, after the split of the *indica* and *japonica* subspecies (Figure 3c). The early branch leading to *indica* and *japonica* was addressed previously [2] and is further corroborated here by 42 *trans* mutations shared among five subgroups. Further support for the phylogeny comes from the distributions of *trans* mutations allocated to certain bins of ten possible combinations of three subgroups and five possible combinations of four subgroups. Of 160 *trans* mutations shared by three subgroups, 107 at 24 loci are in the combinations of *japonica* (*sensu stricto*), *tropical japonica*, and *aromatic*, and three at three loci are shared among *indica* (*sensu stricto*), *aus*, and *aromatic*. One combination, *indica* (*sensu stricto*), *japonica* (*sensu stricto*), and *tropical japonica*, collected 40 *trans* mutations at 11 loci. This pattern is largely a continuation of the early mutations. Of the 32 *trans* mutations shared by four subgroups, 30 are seen in the combination of *indica* (*sensu stricto*), *japonica* (*sensu stricto*), *tropical japonica*, and *aromatic*, with none or one mutation in the other four combinations.

The phylogeny (Figure 3c) indicates that *aus*, *tropical japonica*, and *aromatic* are subgroups that emerged at the later stage of domestication of Asian rice. Given the previous estimate on the duration of the later-stage domestication [2], it is inferred that selection events leading to *aus*, *tropical japonica*, and *aromatic* took place within the last 4–5 millennia in the order shown.

### 3.6. Validity of Mutation Identification

To check the error rate of the mutations identified above, independent data of 82 sequences were sampled from the NCBI database (accessed during the period 14–18 April 2023) for seven genes (*SH4*, *Hd1*, *DFR*, *Hd3a*, *CHS*, *Rc*, and *SPL16*). Alignments of homologous sequences indicate that 26 *Os* mutations fall in the overlapping regions between the two data sets (the data used for identification and those for testing). The test data are consistent with 24 of the 26 mutations (Table S4), with one at the 5′ region of *SH4* being dubious and one at the 5′ region of *Rc* not supported. This result supports that at least 92% of mutations were identified correctly.

## 4. Discussion

### 4.1. GGM-Based Analysis and Specific Test for Asian Rice

Phylogeny reconstruction is mostly dependent on similarity matrices. While valid for evaluating genetic diversity, the prevailing methods (such as SNP-based analyses) have their limitations in handling a complex phylogeny when hybridization and recombination occur. Unlike SNPs-based methods that exclude indels, the GGM-based analysis uses all types of variations and stratifies them to extract phylogenetic relationships among taxa. It does not depend on a matrix of similarity, avoids restrictions on life-history traits, and thus is suitable for many organisms for its enhanced statistical power in inference making in the era of genomics.

The example here shows that although *trans* mutations are present at less than half of the genes surveyed, they are sufficient for phylogenetic inference in Asian rice. The sensitivity of the analysis may vary with other organisms with different selection and/or evolutionary history. It requires an incremental sampling of genes across genomes or increasing genomes in order to attain a sufficient power for phylogeny reconstruction. Identification of recombinant alleles at different loci can further validate the hybridized origin of a relevant taxon. In comparison with these steps generally applicable to other organisms, model-based statistical tests on inner branches, as used here for Asian rice, require some conditions including a relatively large N and a steady environment and selection intensity. When these conditions are not closely met, simulation studies can be conducted to evaluate the bias of using the specific test. Alternatively, different models can be engaged to test the relative lengths of inner branches. The GGM-based method, as shown here, is able to solve issues of complex phylogeny that includes hybridization and recombination. These two processes occur hand-in-hand and are frequently observed in domesticated plants and animals but are typically recalcitrant to the conventional methods of phylogeny reconstruction.

### 4.2. The Early Rice Laid the Foundation for All Subgroups of Asian Rice

Rice domestication is a process of constant fixation of mutations and recombinants favored by past breeders. Passing down of early mutants to later subgroups is more than random sorting but carries phylogenetic information associated with selection (natural and artificial). For *O*. *sativa*, *trans* mutations shared in all five subgroups are most likely from early mutations that were fixed or occurred at high frequencies, and their distributions can be little altered by later practices of breeding (excluding gene editing). For early mutations that were not of high frequency, the tendency to retain the ancestral alleles in *aus* and/or the random (nonrandom) loss of mutations from the other two subgroups (*tropical japonica* and *aromatic*) could lead to a smaller number of shared mutations among the five subgroups (42 at 18 loci) than the number of *trans* mutations (91 at 30 loci) cited earlier between *indica* and *japonica* [2].

Compared with the early mutations, later mutations which emerged after the split of *indica* and *japonica*, may have a different fate. Those specific to the subgroup can be still transient, thus easily subject to random (or nonrandom) loss. Meanwhile, a later differentiated group generally shows fewer loci altered by additional human selection and also fewer varieties than those of an earlier group due to its short history. Since the varietal pattern itself can be easily influenced by the size of cultivation areas and/or replacements by modern hybrids, mutations from recent selection are most vulnerable in modern agriculture, which features mass production of a small number of varieties.

Geographically, the early rice that existed 4–5 millennia ago was found mainly in regions of the lower and central Yangtze River [42]. The later stage of rice domestication clearly involved much broader regions, starting from the split of *indica* (*sensu lato*) and *japonica* (*sensu lato*) in eastern Asia to more southern and northern areas to local selections mostly in southern Asia, some of which have been revealed lately [43]. Archeological evidence indicates that rice cultivation possibly reached south/south east parts of Asia about 4–5 millennia ago [44,45]. Signals of recombination here suggest that a specific member of *tropical japonica* (Chao-Meo type) was likely one of the immediate ancestors of *aromatic*. Consistent with the scenarios above, Chinese germplasm of rice consists of varieties primarily of *indica* and *japonica*, lacks *aus* and *aromatic* and includes *tropical japonica* at a frequency lower than germplasm in the tropical regions [46].

### 4.3. Subgroup Aus Branched off Early in Indica

Since *aus* possesses not only the early mutations of rice but also *trans* mutations specifically shared with *indica* (*sensu stricto*), its root in *indica* is well supported. Its earlier exposure to selection as an independent population made it possible for some transient mutations from early rice to be excluded from its lineage by chance and/or selection. Meanwhile, a significant number of its loci have been under lineage-specific selection. Both processes can cause its distinctiveness, as shown previously [10,18]. The statistical tests on mutation density, which confirm that the branch leading to *aus* and *indica* (*sensu stricto*) is shorter than that leading to *tropical japonica* and *japonica* (*sensu stricto*), simply recapture the pattern of early divergence of *aus*. Although transient *trans* mutations, which were expected to be in a minority, could exist across inner branches, their effect on the statistical tests is benign or negligible when the two branches compared have a recent shared history, as in the cases here with *aus* and *indica* (*sensu stricto*) or *tropical japonica* and *japonica* (*sensu stricto*). This is because the common history (the early rice period) provided the same genetic background, which consequently led to similar proportions of transient mutations in the branches.

Since *aus* is distributed mainly in the subcontinent of India and is hardly seen in Chinese germplasm, its emergence most likely occurred after *indica* reached south and west Asia. The early breeders of *aus* were possibly local to the Himalaya hills [47], whose ancestors or traders could carry early *indica* to the region. Because *O*. *nivara*, one of the wild progenitors of *O*. *sativa*, is tolerant of dry environments, many of its alleles could be passed to *aus* via early *indica* during local selection. Rice strains selected to be capable of growing in harsh environments gave *aus* its characteristic features, i.e., some genomic regions of *aus* are closer to those of *O*. *nivara* than to those of the present day *indica* (*sensu stricto*). This can lead to an erroneous result on its origin under a similarity-based analysis.

At a much later time (about twice longer than the time leading to the separation of *aus* from *indica*), *japonica* was introduced to southeast/south Asia, possibly by different groups of people, leading to the formation of *tropical japonica*. The selection on early *tropical japonica* most likely occurred in a tropical environment on the existing variation in *japonica*. The specifics of these local selections need more investigation.

### 4.4. Aromatic Rice as Hybrid Progeny of Aus and Tropical japonica

Though a principal component analysis [10] already indicated that features of *aromatic* were somewhat between *aus* and *japonica* and a subsequent analysis suggested that *aromatic* could be a hybrid between *aus* and *japonica* [18], the phylogenetic relationship between *aromatic* and *tropical japonica* was unclear until this study. While mutations specific to *tropical japonica* or *aus* are found in *aromatic* genomes, few traces of *japonica* (*sensu stricto*) are seen in *aromatic* here, which diminishes its direct role in the formation of *aromatic* rice. The recombinants of *MYB3* or *Hd1* in the *aromatic* genome clearly support the event of hybridization between *aus* and *tropical japonica*, and more recombinants will be found in future. The shared genomic regions between *aromatic* and *aus* [21] are clearly a consequence of a hybridization event. Incidentally, the previously suspected factor for the aroma, a nonfunctional allele (*badh2.1*) of *BADH2*, is absent in the two *aus* genomes here. Its presence in some accessions of both *aus* and *tropical japonica* [32] is congruent with the hybrid origin of *aromatic*, indicating that the allele is older than the lineage of *aromatic* itself. Since human selection on early *aromatic* could involve more than a specific allele/locus, for instance, gene combinations or recombinants, the lack of selection signal on *BADH2* is not all surprising; instead, other loci with *aromatic*-specific alleles (e.g., *MYB3*, *TCP19*, *MYC2*) should be explored, not only for understanding of selection on *aromatic* but also for their influence on the trait of rice aroma.

Geographically, the selection on early *aromatic* must have occurred in an area where both cultivations of *aus* and *tropical japonica* co-existed at the time. As traditional *aromatic* varieties were mainly from the Himalayan foothills of the Indian subcontinent [48,49], an area which largely overlaps with the current distribution of *aus* [50], early *aromatic* was likely confined to the Indian subcontinent at its incipient stage. As for *aus* and *tropical japonica*, the human history associated with lineage-specific selection on *aromatic* requires more studies.

### 4.5. Applications and Error Rates

Since conventional reconstruction of phylogeny requires data without the effects of selection and/or recombination, only small fragments of genomes (e.g., intergenic regions) are considered suitable. When other regions are used, they need to be tested to confirm they are free of selection or recombination prior to being used. A modern phylogenomic approach can use entire genomic sequences or whole gene sets to leverage regional signals but ignore the heterogeneity of SNPs and indels. The method presented here has none of the above restrictions; thus, it is applicable to all regions of a nuclear genome in theory. The key requirement of the GGM-based method is being able to identify mutations reliably. This requirement may not be met for cases when genomes of immediate ancestor(s) are not available or can be approximated as in rice, which is common. With increasing genomic data becoming available in the future, this limitation can be gradually removed.

For rice, the original parents are no longer extant, but the species (*Oryza rufipogon* and *O*. *nivara*) that contributed the parental individuals [2] about 8–10 kyr ago [6] still exist. Genomes of the wild species can serve as proxies of the original parents with only a small number of errors introduced. This is because the new mutations emerging in the wild relatives over the last 8–10 kyr are in a minority compared with the historical mutations that accumulated in and separated *O. rufipogon* and *O*. *nivara* in the period estimated to be from ~160 kyr [51] to about 340–380 kyr [52]. Even when mutations did occur in a gene in the wild relatives during the past 8–10 kyr, the likelihood of the orthologous gene in rice having the same mutations by chance is minimal. Part of the reason is that the different evolutionary histories of parental species and rice would have impacts on different loci, causing varied distributions of mutations across the genomes. This pattern can significantly lower the error rate of misidentification of a mutation.

Two additional errors, however, may emerge. One is that a mutation identified is not new in rice but inherited from the ancestors and happens to be absent in the current genomes referenced. This error may inflate the number of mutations specific to rice. The probability of such errors is, however, low since the probability for a site to remain unchanged is high for closely related *O*. *rufipogon* and *O*. *nivara*. For instance, if the substitution rate is about 5 × 10^−8^ per site per year in *Oryza*, the probability of two orthologs being identical at a site is 0.98 after 160 kyr or 0.95 after 380 kyr under the Jukes–Cantor model when no selection is involved. Positive selection reduces the probability, while negative selection increases it. The generally low levels of genetic polymorphism between closely related taxa hardly require a specific model of substitutions since similar results are given by the known substitution models during their early divergence.

Another error is taking transient mutations as fixed ones, with the result that comparisons of numbers of presumably fixed mutations between branches can be less accurate. For phylogenetic reconstruction, fixed (or frequent) mutations are preferred over transient (rare) mutations because they contribute more steadily to the physical divergence than the latter. The probability of such errors is, however, smaller for mutations on inner branches than those on the terminal ones. Because of high frequencies, fixed mutations are highly represented in inner branches and also in comparisons between species when each species is represented by one genome. Depending on the level of genetic polymorphism of a taxon, this error is not necessarily reduced by including a few more genomes. In all cases, the error rate of misidentification of mutations can be evaluated with additional sequences, particularly from parental species.

Lastly, the short history of rice implies that repeated mutations at the same nucleotide site of rice are most unlikely, which suggests that nearly all mutations are original, rather than secondary, in rice. This feature permits the simple permutation analysis on *trans* mutations among subgroups here.

Because phylogenetic relationships have a genome-wide influence on all taxa, sampling of loci regardless of chromosome, function, location, or size of a gene permits the phylogenetic relationships to be revealed from a manageable sample size. Here, genes showing epistasis should be sampled only once to reduce bias from correlated mutations. Genes from the same biochemical pathway, however, can be sampled more than once to reflect possibly diverse selection, as seen at the loci of *Asr1* and *Asr2* of the abscisic acid pathway in the common bean [53]. Larger sample sizes can possibly reduce errors due to experiments or complex gene–phenotype relationships but are unlikely to overturn the branching pattern already shown at the phylogenetically informative loci.

### 4.6. Implications of the Phylogeny

With clarified phylogeny, the classification of five subgroups of Asian rice [5] can easily accept new additions. For instance, Madagascar was the latest area in the Old World to grow Asian rice, with subgroups *aus* and *tropical japonica* introduced as the initial major types. Selection on interbred progeny has contributed to the local diversity of rice in the region [54], which can be better understood with the phylogeny. In Africa and America, selection on rice tends to be more environmentally oriented [55,56]. In China, breeders have focused on taking advantage of hybrid vigor. Knowing how rice domestication has shaped its biodiversity over the past millennia helps future planning of healthy agriculture, as much can be learnt from early breeders.

The GGM-based method can be integrated into a broad application that targets uncovering key alleles/mutations that affect traits of interest such as tolerance to stress [57]. When a package that can reliably delineate gene regions on a selected number of genes and integrate all mutations is provided in a phylogenomic program, the MMG method can readily stratify mutations and use their temporal distributions to build a phylogeny or test for or against candidate phylogenetic trees inferred from other methods. For complex phylogenies, the MMG method can rebuild the past history alone when the data have a sufficient power, providing a valid solution for phylogeny reconstruction, as shown here. In future, an MMG-based phylogeny could be statistically tested by a program using approximate Bayesian computation when all mutations are used by machine-learning to generate a valid distribution of random mutations among branches.

## 5. Conclusions

Knowing the domestication history of rice can benefit targeted research on food shortages and nutrition, as well as provide temporal coordinates in understanding past human migration and development. The phylogenetic relationships among the five subgroups were clarified in this study; evidently, after much selection during the phase I domestication of rice [2], significant events of human selection led to the emergence of *aus*, *tropical japonica*, and *aromatic* in the phase-II domestication of rice. Cultivation of Asian rice should be continued in the direction of improved biodiversity, with the purpose of ensuring that the crop remains healthy for generations to come.

## Figures and Tables

**Table 1:** A list of 51 loci showing mutations informative to relationships of the subgroups of Asian rice.

Chromosome	Gene ^a^	Length (bp) ^b^		Early Mutations ^c^	Mutation Density ^d^		Mutations Specific to Two Subgroups					
							*aus* and *indica* ^e^	Mutation Density		*tropical japonica* and *japonica* ^f^	Mutation Density ^g^	
		5′	Coding		5′	Coding		5′	Coding		5′	Coding
1	*CKX2*	1021	1704	0 (5′), 1 (coding)			4 (5′), 1 (coding)	0.0039	0.0006	2 (5′), 0 (coding) ^f^	0.0020	
	*DFR*	1036	1119	**1 (5′)**, 1 (5′), 0 (coding)	0.0010							
	*NOG1*	1012	1170							20 (5′), 1 (coding) ^f^	0.0198	0.0009
	*ERF4a ^a^*	1090	708				3 (5′), 1 (coding)	0.0028	0.0014			
	*LAC7 ^a^*	1025	1680				0 (5′), 1 (coding)		0.0006	2 (5′), 2 (coding) ^f^	0.0020	0.0012
	*SD1*	1004	1170	0 (5′), **1 (coding)**		0.0009	0 (5′), 1 (coding)		0.0009	4 (5′), 2 (coding) ^f^	0.0040	0.0017
2	*FUWA ^a^*	1028	1482				1 (5′), 0 (coding) ^e^	0.0010		10 (5′), 0 (coding)	0.0097	
	*GW2 ^a^*	1020	1278							8 (5′), 0 (coding)	0.0078	
	*SK2*	1013	1029	**1 (5′)**, 1 (5′), 0 (coding)	0.0010					7 (5′), 1 (coding) ^f^	0.0069	0.0010
3	*SUS4*	1057	2430							4 (5′), 1 (coding) ^f^	0.0038	0.0004
	*MYB3*	1017	1053	0 (5′), 2 (coding)								
	*GL3.2*	1153	1554	**1 (5′)**, 3 (5′), 3 (coding)	0.0009		1 (5′), 0 (coding)	0.0009				
	*TAC3 ^a^*	1024	459				1 (5′), 0 (coding) ^e^	0.0010		1 (5′), 0 (coding)	0.0010	
	*Hd6*	2280	627	14 (5′), 0 (coding)								
	*Dst*	1031	927							3 (5′), 3 (coding) ^f^	0.0029	0.0032
	*CHI*	1400	762	3 (5′), 1 (coding)			0 (5′), 1 (coding)		0.0013	5 (5′), 0 (coding) ^f^	0.0036	
4	*GIF1*	1238	1794	2 (5′), 3 (**1 coding**)		0.0006						
	*Myb4*	1008	789	**3 (5′)**, **3 (coding)**	0.0030	0.0038						
	*An-2*	1000	753	**4 (5′)**, 0 (coding)	0.0040							
	*unknown*	1010	1695	0 (5′), **1 (coding)**		0.0006				1 (5′), 2 (coding) ^f^	0.0010	0.0012
	*AGO2*	1094	3123	2 (5′), **1 (coding)**		0.0003				4 (5′), 6 (coding) ^f^	0.0037	0.0019
	*IPK1 ^a^*	1000	1338				0 (5′), 1 (coding)		0.0007	2 (5′), 0 (coding) ^f^	0.0020	
	*F3H*	1002	1134				3 (5′), 1 (coding)	0.0030	0.0009	3 (5′), 4 (coding) ^f^	0.0030	0.0035
	*SH4*	1006	1173	**5 (5′)**, 1 (5′), **1 (coding)**	0.0050	0.0009				1 (5′), 0 (coding)	0.0010	
5	*GS5*	1010	1458	1 (5′), 1 (coding)						1 (5′), 2 (coding) ^f^	0.0010	0.0014
	*ACS3*	1000	1314							1 (5′), 0 (coding) ^f^	0.0010	
	*SH5*	1466	1743							3 (5′), 3 (coding) ^f^	0.0020	0.0017
	*T6P (TPS1)*	1018	2595							5 (5′), 4 (coding) ^f^	0.0049	0.0015
6	*EPSPS*	1007	1548	3 (5′), 0 (coding)			0 (5′), 1 (coding)		0.0006			
	*Hd3a*	1023	540	4 (5′), 0 (coding)						0 (5′), 2 (coding)		0.0037
	*C1*	1002	819	**2 (5′)**, 0 (coding)	0.0020							
	*TCP19*	1116	1209	**1 (5′)**, 1 (5′), 0 (coding)	0.0009					1 (5′), 0 (coding)	0.0009	
	*Hd1*	1375	1351	1 (5′), 2 **(1 coding)**		0.0007						
7	*PROG1*	1000	504	**1 (5′)**, 1 (5′), **1 (coding)**	0.0010	0.0020						
	*Rc*	594	2013	**1 (5′)**, 2 (5′), 3 (coding)	0.0017		1 (5′), 0 (coding)	0.0017				
	*SPL13 ^a^*	1014	651				1 (5′), 0 (coding) ^e^	0.0010				
	*WG7 ^a^*	1064	4803	8 (5′), 4 (coding)								
8	*RAE2*	1003	593	**3 (5′)**, **1 (coding)**	0.0030	0.0017						
	*SPL16*	1017	1368	3 (5′), 0 (coding)						1 (5′), 1 (coding) ^f^	0.0010	0.0007
9	*unknown*	1075	360							4 (5′), 2 (coding) ^f^	0.0037	0.0056
	*DEP1*	1000	1281				0 (5′), 2 (coding)		0.0016	4 (5′), 0 (coding) ^f^	0.0040	
	*PGI*	1000	1878							2 (5′), 0 (coding)	0.0020	
	*PRR95*	1034	1872	**1 (5′)**, 1 (coding)	0.0010					1 (5′), 1 (coding) ^f^	0.0010	0.0005
	*DHQS*	1013	1332							1 (5′), 0 (coding) ^f^	0.0010	
10	*DAHPS2*	1007	1509	**5 (5′)**, **1 (coding)**	0.0050	0.0007						
	*MYC2*	1015	2261				1 (5′), 0 (coding)	0.0010		2 (5′), 0 (coding) ^f^	0.0020	
11	*PK1*	1016	1584	2 (5′), 0 (coding)						6 (5′), 0 (coding)	0.0059	
	*unknown*	1004	1782							8 (5′), 0 (coding)	0.0080	
	*CHS*	1254	1197				1 (5′), 2 (coding)	0.0008	0.0017	1 (5′), 0 (coding)	0.0008	
12	*Pi-ta ^a^*	1277	2787							1 (5′), 0 (coding)	0.0008	
	*unknown*	1013	1348	2 (5′), **3 (coding)**		0.0022						

Note: ^a^ Locus not included in Lu et al., 2022. [2] The underlined ones were under positive selection during the early stage of domestication, as shown in Lu et al., 2022. [2] ^b^ The length is for the alignment, with 5′-region not including ATG and coding regions having the stop codon counted. ^c^ Mutations specific to *O*. *sativa* and shared between at least *indica* and *japonica* (bold ones are shared among all five subgroups). ^d^ Mutation density was computed for the case of five-subgroup sharing only. ^e^ Mutations shared also with aromatic are marked in e. ^f^ Mutations shared also with aromatic are marked in f. ^g^ Mutation density is in the unit of mutation number per nucleotide of a gene region (5′ or coding) per branch shared between two subgroups.

**Table 2:** Gene regions showing specific mutations shared by *aromatic* and *aus* or *tropical japonica*.

Subgroups	Gene 1 ^a^	Gene 2	Gene 3	Gene 4	Gene 5	Gene 6	Gene 7	Gene 8
*aus*-*aromatic*	*GIF1* 5′(2)	*RAE2* cds (3) ^b^	*FUWA* 5′(5)	*TAC3* 5′(1) ^c^	*SPL13*-5′ (1) ^c^			
	*GIF1* cds (1)		*FUWA* cds (1)					
*tropical*-*aromatic*	*CKX2* 5′(1)	*DFR* 5′(12)	*Hd3a* 5′(3)	*Hd1* 5′(1)	*Rc* 5′(1)	*SK2* cds (1)	*PRR95* cds (1)	Os09g26890 5′(1)
	*CKX2* cds (1)							

Note: ^a^ Number of *trans* mutations are in the parentheses. ^b^ The mutations are transient in *indica* (*sensu stricto*). ^c^ The mutation is transient in *japonica* (*sensu stricto*).

## Data Availability

All data generated or analyzed during this study are included in this publication.

## Supplementary Materials

The following supporting information can be downloaded at: https://www.mdpi.com/article/10.3390/genes14071412/s1. Figure S1: Identification of rice mutations across 51 genes; Figure S2: The coding regions of *OsHd1* compared among three subgroups; Figure S3: Mutation distributions at the locus *BADH2* (Os08g32870.1); Table S1: Genomes analyzed in this study; Table S2: List of 121 *Oryza* genes surveyed in this study; Table S3: Information on the 51 *Os* loci with *trans* mutations; Table S4: An examination of *Os* mutations with additional 82 NCBI entries. References [58,59,60,61,62,63,64,65,66,67,68,69,70,71,72,73,74,75,76,77,78,79,80,81,82,83,84,85,86,87,88,89,90,91,92,93,94,95,96,97,98,99,100,101,102,103] are cited in the supplementary materials.
