# Supplementary material for: Gene Genealogy-Based Mutation Analysis Reveals Emergence of Aus, Tropical japonica, and Aromatic of Oryza sativa during the Later Stage of Rice Domestication
Source: Genes (Basel). 2023 Jul 8;14(7):1412. doi: 10.3390/genes14071412 (PMC10379336; doi:10.3390/genes14071412)
Supplement: Supplementary file 1 [file genes-14-01412-s001.zip › genes-2404707-supplementary.pdf]

Supplementary Information to

Gene-genealogy-based mutation analysis reveals emergence of *aus*, *tropical japonica*, and *aromatic* of *Oryza sativa* during the later stage of rice domestication

Yingqing Lu[1,2]

[1]State Key Laboratory of Systematic and Evolutionary Botany, Institute of Botany, Chinese Academy of Sciences, 20 Nan Xin Cun, Beijing 100093, China

[2]University of Chinese Academy of Sciences, Beijing 100049, China

Figure S1. Identification of rice mutations across 51 sampled genes. **a.** Chromosome 1. **b.** Chromosome 2. **c.** Chromosome 3. **d.** Chromosome 4. **e.** Chromosome 5. **f.** Chromosome 6. **g.** Chromosome 7. **h.** Chromosome 8. **i.** Chromosome 9. **j.** Chromosome 10. **k.** Chromosome 11. **l.** Chromosome 12.

Figure S2. The coding regions of *OsHd1* compared among three subgroups.

Figure S3. Mutation distributions at the locus *BADH2* (Os08g32870.1).

Table S1. Genomes analyzed in this study.

Table S2. List of 121 *Oryza* genes surveyed in this study.

Table S3. Information on the 51 *Os* loci with *trans* mutations.

Table S4. An examination of *Os* mutations with additional 82 NCBI entries.

Table S1. Genomes analyzed in this study.

Species	Subgroup	Variety	Accession[a]	Submitter	Genome coverage[b]
O. rufipogon		W1943	PRJEB4137[a]	NCGR	130x
O. nivara		IRGC 100897	PRJNA48107[a]	Arizona Genomics Institute	102x
O. sativa	*aus*	Netal Boro	PRJNA565483[a]	University of Arizona	111x
		N22	PRJNA315689	University of Arizona	65x[b]
	tropical japonica	Ketan Nangka	PRJNA564615[a]	University of Arizona	125x
		Chao Meo	PRJNA565484	University of Arizona	123x
	japonica	Nipponbare	PRJNA12269[a]	National Institute of Agricultural Sciences	~100x
		Kitaake	PRJNA448171	DOE Joint Genome Institute	532x
	indica	9311	PRJNA427873[a]	Chinese Academy of Agricultural Sciences	116x
		Shuhui498	PRJNA318714	Chinese Academy of Sciences	120x
		Minghui63	PRJNA30254	Huazhong Agricultural University	120x
	aromatic	IRGC 12485-1	PRJNA565479a	University of Arizona	112x

[a] accessions included in the alignments of all genes, except noted otherwise. [b] used only when needed for rechecking of mutation

Table S2 List of 121 *Oryza* genes surveyed in this study.

Chromosome	Gene	RAP ID†	MSU ID	Note
1	*ME*	Os01g0188400	Os01g09320.1	
	CKX2	Os01g0197700	Os01g10110.1	
	ANS1	Os01g0372500	Os01g27490.1	
	LAC1		Os01g27700.1	
	DFR	Os01g0633500	Os01g44260.1	
	NOG1	Os01g0752200	Os01g54860.1	
	CM3	Os01g0764400	Os01g55870.1	
	ERF4a	Os01g0797600	Os01g58420.1	
	iPGAM1	Os01g0817700	Os01g60190.1	
	qSH1	Os01g0848400	Os01g62920.1	
	LAC7	Os01g0850700	Os01g63190.1	
	SD1	Os01g0883800	Os01g66100.1	
	BG3		Os01g48800.1	
2	*DHD4*		Os02g01990.1	
	FUWA	Os02g0234200	Os02g13950.1	
	GW2		Os02g14720.1	
	SBE3	Os02g0528200	Os02g32660.1	
	ZB8	Os02g0627100	Os02g41680.1	
	PAC1	Os02g0682500	Os02g45810.2	
	SK2	Os02g0687500	Os02g46220.1	
	FLS	Os02g0767300	Os02g52840.1	
	DTH2	Os02g0724000	Os02g49230.1	
3	*MADS1*	Os03g0215400	Os03g11614.1	
	CS	not listed	Os03g14990.1	
	LAR	Os03g0259400	Os03g15360.2	
	Unknown		Os03g16610.1	
	iPGAM2	Os03g0330200	Os03g21260.1	
	SUS2(4)	Os03g0340500	Os03g22120.2	
	DAHPS1	Os03g0389700	Os03g27230.1	
	GS3	Os03g0407400	not listed	
	OsMYB3	Os03g0410000	Os03g29614.1	
	GL3.2	Os03g0417700	Os03g30420.1	
	TAC3	Os03g0726700	Os03g51660.1	
	TB1	Os03g0706500	Os03g49880.1	
	Hd6	Os03g0762000	Os03g55389.1	
	Dst	Os03g0786400	Os03g57240.1	
	CHI	Os03g0819600	Os03g60509.1	
	ASA1	Os03g0826500	Os03g61120.1	
4	*An-1*	Os04g0350700	Os04g28280.2	
	GIF1	Os04g0413500	Os04g33740.1	
	MYB15	Os04g0517100	Os04g43680.1	
	An-2	Os04g0518800	Os04g43840.1	
	Unknown	Os04g0557200	Os04g47040.1	
	B2	Os04g0557500	Os04g47059.1	
	B1	Os04g0557800	Os04g47080.1	
	AGO2	Os04g0615700	Os04g52540.1	
	IPK1	Os04g0661200	Os04g56580.1	
	F3H	Os04g0662600	Os04g56700.1	
	SH4	Os04g0670900	Os04g57530.1	
	PK3	Os04g0677500	Os04g58110.1	
	SHAT1	Os04g0649100	Os04g55560.1	
5	*Chalk5*	Os05g0156900	Os05g06480.1	
	GS5	Os05g0158500	Os05g06660.1	
	qSW5	Os05g0187500	Os05g09520.1	
	unknown	Os05g0196600	Os05g10780.1	*ACS3*
	ACC7	Os05g0319200	Os05g25490.1	
	C4H	Os05g0320700	Os05g25640.1	
	FBX165		Os05g25580.1	
	SH5	Os05g0455200	Os05g38120.1	
	T6P (TPS1)	Os05g0518600	Os05g44210.1	
6	*EPSPS*	Os06g0133900	Os06g04280.1	
	Hd3a	Os06g0157700	Os06g06320.1	
	SSY1	Os06g0160700	Os06g06560.1	
	C1	Os06g0205000	Os06g10340.1	
	TCP19	Os06g0226700	Os06g12230.1	
	Hd1	Os06g0275000	Os06g16370.1	
	3GT	Os06g0291100	Os06g18790.1	
	vATPB1	Os06g0568200	Os06g37180.1	
	GL6	Os06g0666100	Os06g45540.1	
7	*PROG1*	Os07g0153600	Os07g05900.1	
	bZIP58	Os07g0182000	Os07g08420.1	
	Rc	Os07g0211500	Os07g11030.1	
	SSH1	Os07g0235800	Os07g13170.1	
	Ghd7	Os07g0261200	Os07g15770.1	
	SPL13	Os07g0505200	Os07g32170.1	
	SDR4	Os07g0585700	Os07g39700.1	
	BG2	Os07g0603700	Os07g41240.1	
	DAHPSp	Os07g0622200	Os07g42960.1	
	NADH	Os07g0645400	Os07g45090.1	
	WG7	Os07g0669800	Os07g47360.1	
	PRR37	Os07g0695100	Os07g49460.1	
	SAP16		Os07g38240.1	
8	*Hd5(Gdh8)*	Os08g0174500	Os08g07740.1	
	SSY3	Os08g0191500	Os08g07740.1	
	APS1	Os08g0345800	Os08g09230.2	
	ARE1		Os08g12780.1	
	BADH2		Os08g32870.1	
	CM4	Os08g0441600	Os08g34290.1	
	RAE2	Os08g0485500	Os08g37890.1	
	IPA1	Os08g0509600	Os08g39890.1	
	SPL16	Os08g0531600	Os08g41940.1	
9	*Sub1B*		Os09g11480.1	
	unknown	Os09g0440600	Os09g26890.1	
	unknown	Os09g0440700	Os09g26900.1	
	DEP1	Os09g0441900	Os09g26999.1	
	PGI	Os09g0465600	Os09g29070.1	
	bZIP73	Os09g0474000	Os09g29820.1	
	PRR95	Os09g0532400	Os09g36220.1	
	DHQS	Os09g0539100	Os09g36800.1	
10	*PGMp*	Os10g0189100	Os10g11140.2	
	F3'H	Os10g0320100	Os10g17260.1	
	Ehd1	Os10g0463400	Os10g32600.1	
	DAHPS2	Os10g0564400	Os10g41480.1	
	MYC2	Os10g0575000	Os10g42430.1	
	NRT1.1B	Os10g0554200	Os10g40600.1	
11	*PK1*	Os11g0148500	Os11g05110.2	
	unknown	Os11g0181100	Os11g07910.1	
	ADH2	Os11g0210500	Os11g10510.1	
	unknown	Os11g0483900	Os11g29350.2	
	unknown	Os11g0484500	Os11g29400.1	
	CHS	Os11g0530600	Os11g32650.1	
	CTS		Os11g40130.1	
	unknown	Os11g0615600		
12	*unknown*	Os12g0108500	Os12g01760.1	
	Pi-ta	Os12g0281300	Os12g18360.1	
	unknown	Os12g0533700	Os12g34860.1	
	SDH2(DHQ D2)	Os12g0534000	Os12g34874.1	
	unknown	Os12g0534700	Os12g34920.1	
	CM2	Os12g0578200	Os12g38900.1	
	unknown	Os12g0578400	Os12g38920.1	
	Pstol1		*MN199305*	

Table S3. Information on the 51 *Os* loci with *trans* mutations.

Chromosome	Gene	RAP ID	MSU ID[a]	Known function[b]	Reference
**1**	*CKX2*	Os01g0197700	Os01g10110.1	Cytokinin oxidase	[58]
	DFR	Os01g0633500	Os01g44260.1	Flavonoid network	[59]
	NOG1	Os01g0752200	Os01g54860.1	Grain number	[60]
	ERF4a	Os01g0797600	Os01g58420.1	Drought tolerance	[61]
	LAC7	Os01g0850700	Os01g63190.1	Laccase, lignification	[62]
	SD1	Os01g0883800	Os01g66100.1	Gibberellin biosynthesis	[63]
**2**	*FUWA*	Os02g0234200	Os02g13950.1	Grain architecture	[64]
	GW2	-	Os02g14720.1	Grain size	[65]
	SK2	Os02g0687500	Os02g46220.1	*Shikimate pathway*	[2]
**3**	*SUS4*	Os03g0340500	Os03g22120.2	Sucrose synthase	[66]
	MYB3	Os03g0410000	Os03g29614.1	Flavonoid network	[67]
	GL3.2	Os03g0417700	Os03g30420.1	Grain development	[68]
	TAC3	Os03g0726700	Os03g51660.1	Tiller angle	[69]
	Hd6	Os03g0762000	Os03g55389.1	Flowering	[70]
	Dst	Os03g0786400	Os03g57240.1	Grain production	[71]
	CHI	Os03g0819600	Os03g60509.1	Flavonoid network	[72]
**4**	*GIF1*	Os04g0413500	Os04g33740.1	Grain filling	[73]
	Myb4	Os04g0517100	Os04g43680.1	*Stress tolerance*	[74]
	An-2	Os04g0518800	Os04g43840.1	Awn length	[75]
	unknown	Os04g0557200	Os04g47040.1	Unknown	This study
	AGO2	Os04g0615700	Os04g52540.1	Grain length/salt tolerance	[76]
	IPK1	Os04g0661200	Os04g56580.1	*Phosphate storage*	[77]
	F3H	Os04g0662600	Os04g56700.1	Flavonoid network	[78]
	SH4	Os04g0670900	Os04g57530.1	Seed shattering	[79]
**5**	*GS5*	Os05g0158500	Os05g06660.1	Grain width	[80]
	ACS3	Os05g0196600	Os05g10780.1	Ethylene biosynthesis	[81]
	SH5	Os05g0455200	Os05g38120.1	Seed shattering	[82]
	T6P (TPS1)	Os05g0518600	Os05g44210.1	*Trehalose-6-phosphate synthase*	[83]
**6**	*EPSPS*	Os06g0133900	Os06g04280.1	Shikimate pathway	[84]
	Hd3a	Os06g0157700	Os06g06320.1	Flowering pathway	[85]
	C1	Os06g0205000	Os06g10340.1	Flavonoid network	[86]
	TCP19	Os06g0226700	Os06g12230.1	Tillering response to nitrogen	[87]
	Hd1	Os06g0275000	Os06g16370.1	Flowering pathway	[88]
**7**	*PROG1*	Os07g0153600	Os07g05900.1	Growth angle	[89]
	Rc	Os07g0211500	Os07g11030.1	Flavonoid network	[90]
	SPL13	Os07g0505200	not listed	Grain size	[91]
	WG7	Os07g0669800	Os07g47360.1	Grain width	[92]
**8**	*RAE2*	Os08g0485500	Os08g37890.1	Awnless	[93]
	SPL16	Os08g0531600	Os08g41940.1	Grain width	[94]
**9**	*unknown*	Os09g0440600	Os09g26890.1	Unknown	This study
	DEP1	Os09g0441900	Os09g26999.1	Grain yield	[95]
	PGI	Os09g0465600	Os09g29070.1	*Vitamin C biosynthesis*	[96]
	PRR95	Os09g0532400	Os09g36220.1	*Circadian clock*	[97]
	DHQS	Os09g0539100	Os09g36800.1	*Shikimate pathway*	[2]
**10**	*DAHPS2*	Os10g0564400	Os10g41480.1	*Shikimate pathway*	[98]
	MYC2	Os10g0575000	Os10g42430.1	Jasmonate regulation	[99]
**11**	*PK1*	Os11g0148500	Os11g05110.2	*Glycolysis*	[100]
	unknown	Os11g0181100	Os11g07910.1	*Transmembrane 9 gene*	[101]
	CHS	Os11g0530600	Os11g32650.1	Flavonoid network	[102]
**12**	*Pi-ta*	Os12g0281300	Os12g18360.1	Blast resistance	[103]
	unknown	Os12g0533700	Os12g34860.1	Unknown	[2]

[a] based on the Nipponbare genome annotated by MSU Rice Genome Annotation Project.[b] known function is from the referenced report,and suspected one in italic.

Table S4. An examination of *Os* mutations with additional 82 NCBI entries.

Gene	NCBI sample	Sample size (type)	Mutation	Error rate
SH4	GX6113b(EU99922),YN3-9(EU999925),VOC4b(EU999927), W630(AB493391), 80506(EU999893), 100916(GU220982),104501(GU220983)	7 *Or*	5’: 4 valid, 1 error;<br>cds: 1 valid	1/6
Hd1	Or-cds: SriL(JN594481), Tw6(JN594490); On-cds: Mya(JN594499), 0106(JN594497)	2 *Or* , 2 *On*	cds: 2 valid	0/2
DFR	Or-cds: NSFTV490(MW310648), NSFTV766(MW310646); On-cds:NSFTV757(MW310647)	2 *Or* , 1 *On*	cds: 1 valid	0/1
Hd3a	Or-5’:W0120, 0547, 1666, 1943, 1944, 2014, 2265; IRGC103827, 104404, 104626, 104714, 106346, 106363; Or-cds: W593, 0120, 0574, 1666, 1806, 1807, 1865,1939,1943,1944,1975,2014,2265; IRGC101508,102171,102179,103827, 104404,104626,104684,104969; MAFF AS017, 062	13 Or (5’), 23 *Or* (cds)	5’: 4 valid;<br>cds: 2 valid	0/6
CHS	japonica: Changxianggeng1813; indica: Zhenshan97, GuangLuAi4(CT828776),	4 *Os*	cds: 2 valid	0/2
Rc	Pop set 300827056	14 *Or* , 2 *On*	5’: 4 valid, 1 dubious;<br>cds: 3 valid	1/8
SPL16	Pop set 371931467	5 *Or* , 5 *On* , 5 *indica* , 10 japonica	cds: 1 valid	0/1
	Total samples	82	Average error rate	2/26 = 0.077

a. Chromosome 1
(67 *trans* mutations)

1 ***CKX2*** (Os01g10110.1)

***CKX2*-5′ (1021 bp alignment)**

Group	22	138	316-	386	427	448	898	1000—
Or	T	G	-	G	T	T	A	C..
On	T	G	-	G	T	T	A	-
aus	A	A	-	A	T	T	G	-
ind	A	A	-	A	T	T	G	-
jap	T	G	C.	G	A	T	A	C.T.
tro	T	G	C.	G	A	A	A	C..
aro	T	G	C.	G	A	A	A	C..

***CKX2*-cds (1704 bp alignment)**

Group	161	226–231	232–237	319	353	1611
Or	C	(GCC)x2	(GCC)x2	G	G	G
On	C	(GCC)x2	(GCC)x2	G	G	G
aus	C	-	-	G	G	G
ind	C	-	-	G	G	G
jap	G	-	(GCC)x2	G	A	T
tro	C	(GCC)x2	(GCC)x2	T	G	G
aro	C	(GCC)x2	(GCC)x2	T	G	G

2 ***DFR*** (Os01g44260.1)

***DFR*-5′(1036 bp alignment)**

Group	1<br>3	2<br>1	4<br>4	4<br>7	59,<br>685	99,<br>739	88,<br>768	125,<br>175,<br>676,<br>793	12<br>9	13<br>2	20<br>6	228,<br>269,<br>271	28<br>3	366,<br>373	477<br>–<br>487	68<br>4	69<br>3	74<br>3	77<br>5	87<br>6
Or	A	G	A	-	-	T	C	G	G	A.	G	A	A	T	T.	-	G	G	G	-
On	A	G	A	-	-	T	C	G	G	A.	G	A	A	T	T.	-	G	G	G	-
aus	A	A	A	-	-	T	C	G	G	-	T	A	A	T	T.	A	A	G	G	-
ind	A	G	A	-	-	T	C	G	G	A.	G	A	A	T	-	A	A	A	G	-
jap	A	A	A	-	-	T	C	G	G	-	T	A	A	T	T.	A	A	G	G	-
tro	G	G	T	T	A	C	T	A	G	A.	G	G	T	C	T.	A	G	A	T	T.
aro	G	G	T	T	A	C	T	A	A	A.	G	G	T	C	T.	A	G	A	T	T.
																	*			

***DFR*- cds (1119 bp alignment)**

Group	164	301	462	661<br>–<br>663	711
Or	C	A	C	T.	C
On	C	A	C	T.	C
aus	C	G	C	T.	C
ind	C	A	T	T.	T
jap	A	G	C	T.	C
tro	C	A	C	T./-	C
aro	C	A	C	T.	C

3 ***NOG1*** (Os01g54860.1)

***NOG1*-5′ (1012 bp alignment)**

Group	6<br>5	7<br>8	2<br>1<br>6	2<br>2<br>5	3<br>0<br>4	3<br>3<br>1	3<br>4<br>9	3<br>6<br>6	4<br>4<br>1	4<br>8<br>1	5<br>2<br>5	5<br>2<br>8	5<br>3<br>8	5<br>7<br>0	5<br>8<br>4	5<br>9<br>5	6<br>0<br>9	6<br>2<br>2	64<br>0	6<br>4<br>9	66<br>7	6<br>8<br>3	7<br>8<br>3	8<br>2<br>7
Or	T	C	G	A	T	C	G	T.	C	G	T	C	G	T	-	G	A	G	CT	C	GT	T	T	T
On	T	C	G	C	T	C	G	T.	C	T	T	C	G	T	-	G	A	G	CT	C	GT	T	T	T
aus	T	C	G	C	T	C	G	-	C	G	T	T	G	T	C	G	A	G	CT	C	GT	T	C	T
ind	T	C	G	C	T	C	G	T.	C	G	T	C	G	T	-	G	A	G	CT	C	GT	T	T	T
jap	A	G	A	T	C	T	T	T.	T	-	A	C	T	A	-	A	G	A	-	T	AA	-	T	C
tro	A	G	A	T	C	T	T	T.	T	-	A	C	T	A	-	A	G	A	-	T	AA	-	T	C
aro	A	G	A	T	C	T	T	T.	T	-	A	C	T	A	-	A	G	A	-	T	AA	-	T	C

***NOG1*-cds (1197 bp alignment)**

Group	66	1063
Or	A	G.
On	A	G.
aus	A	G.
ind	A	-
jap	C	G.
tro	C	G.
aro	C	G.

4 ***ERF4a*** (Os01g58420.1)

***ERF4a*-5′ (1139 bp alignment)**

Group	27,28	242	273	274,275
Or	CG	A	T	-
On	CG	A	A	-
aus	TT	T	A	AA
ind	TT/CG	T/A	-/T	AA/-
jap	CG	A	T	-
tro	CG	A	T	-
aro	CG	A	T	-

***ERF4a*-cds (708 bp alignment)**

Group	492
Or	T
On	T
aus	A
ind	A/T
jap	T
tro	T
aro	T

5 ***LAC7*** (Os01g63190.1)

***LAC7*-5′ (1025 bp alignment)**

Group	491	583
Or	G	A
On	G	A
aus	G	A
ind	G	A
jap	A	T
tro	A	T
aro	A	T

***LAC7*-cds (1680 bp alignment)**

Group	170	753	935
Or	A	C	G
On	A	C	G
aus	A	T	G
ind	A	T	G
jap	T	C	A
tro	T	C	A
aro	T	C	A

6 ***SD1*** (Os01g66100.1)

***SD1*-5′ (1004 bp alignment)**

Group	18	194	217	242	304	337	617	621	648	731	829
Or	G	G	A	C	A	A	T	C	C	C	C
On	G	G	A	C	A	A	T	C	G	C	C
aus	G	A	G	T	T	**A**	A	T	G	C	A
ind	G	G	A	C	A	A	T	C	G	C	C
jap	T	G	A	C	A	T	T	C	A	T	C
tro	T	G	A	C	A	T	T	C	A	T	C
aro	T	G	A	C	A	T	T	C	A	T	C

***SD1*-cds (1170 bp alignment)**

Group	297	299	654	844-5	988	1019
Or	C..	G	C	GG	-	-
On	C..	G	C	GG	-	-
aus	C..	G	C	-	G	G
ind	-/C..	-/G	T	GG	-/G	G
jap	C..	A	C	GG	G	A
tro	C..	A	C	GG	G	A
aro	C..	A	C	GG	G	A
					*	

1

b. Chromosome 2
(38 *trans* mutations)

7 ***FUWA*** (Os02g13950)

***FUWA*-5′ (1049 bp alignment)**

Group	21	202	222	264	309	395	431	441	496	514	569	574	582	583	631	659	706
Or	G	-	G	G	T	-	A	-	A	C	T	T	G	T	T	C	A
On	G	-	G	G	T	-	A	-	A	C	T	G	G	T	C	C	A
aus	G	-	A	G	T	-	T	-	A	C	T	A	A	C	C	T	A
ind	G	-	G	G	T	-	A	-	A	C	T	G	G	C	C	C	A
jap	A	C.	G	A	A	A	A	T	T	T	C	G	G	T	T	C	G
tro	A	C.	G	A	A	A	A	T	T	T	C	G	G	T	T	C	G
aro	G	-	A	G	T	-	T	-	A	C	T	A	A	C	C	T	A

***FUWA*-cds (1482 bp alignment)**

Group	876
Or	G
On	G
aus	T
ind	G
jap	G
tro	G
aro	T

8 ***GW2* (**Os02g14720.1**)**

***GW2*-5′ (1020 bp alignment)**

Group	9	25	30	116	150	158	185	244	306	418	611—619	709	787	796	949
Or	G	-	-	G	A	G	G..	A	G	G	CAG	G	G	T	-
On	G	4C	T	G	A	G	G..	A	G	G	CAG	G	A	G	-
aus	-	3C	T	G	A	G	G..	A	G	G	CAG	G	A	G	-
ind	G	3-5C	-/C	G	A	G	G..	A	G	G	CAG/-	G	A	A/G	-
jap	G	3C	-	A	T	G	--	G	T	A	(CAG)x3	A	-	A	-
tro	G	5C	-	A	A	A	--	G	T	A	(CAG)x2	A	-	A	AGG
aro	G	5C	T	G	A	G	G..	A	G	G	CAG	G	A	G	-

***GW2*-cds (1278 bp alignment)**

Group	114
Or	C
On	C
aus	C
ind	T
jap	C
tro	C
aro	C

9 ***SK2* (**Os02g46220.1**)**

***SK2*-5′ (1013 bp alignment)**

Group	17	61	364	428	634	718	733	734	987
Or	C	A	T	C	A	-	C	G	-
On	C	A	T	C	A	T	C	G	-
aus	C	A	T	C	A	C	C	G	-
ind	C	A	T	C	A	C	C	A	-
jap	T	G	C	T	G	C	T	A	TCGCC
tro	T	G	C	T	G	C	T	A	TCGCC
aro	T	G	C	T	G	C	T	A	TCGCC

*

***SK2*-cds (1029 bp alignment)**

Group	48, 49	372
Or	AG	A
On	AG	A
aus	AG	A
ind	AG	A
jap	AG	G
tro	--	G
aro	--	G

c. Chromosome 3
(50 *trans* mutations)

10 *SUS4* (Os03g22120.2)

***SUS4*-5′ (1057 bp alignment)**

Group	2	10	459	689
Or	G	C	T	C
On	G	C	T	C
aus	G	C	T	C
ind	G	C	T	C
jap	A	T	A	A
tro	A	T	A	A
aro	A	T	A	A

***SUS4*-cds (2430 bp alignment)**

Group	336	490	2227
Or	G	T.	C
On	G	T.	C
aus	G	-	C
ind	A	T.	C
jap	G	T.	A
tro	G	T.	A
aro	G	T.	A

11 *MYB3* (Os03g29614.1)

***MYB3*-5′ (1017 bp alignment)**

Group	133	135	139	154	184	271	331	344	368	612—	685	703	712	937	955	983
Or	C	T	A	G	G.	C	G	C	G	C.	G	G	C	AA	GA	G.
On	C	T	A	G	G.	C	G	C	G	C.T.	G	G	C	AA	GA	G.
aus	C	T	A	G	G.	C	G	C	G	C.T.	G	G	C	AA	GA	G.
ind	C	T	A	G	G.	C	G	C	G	C.	G	G	C	AA	GA	G.
jap	C	T	A	G	G.	C	G	C	G	C.	G	G	C	AA	GA	G.
tro	C	T	A	G	G.	C	G	C	G	C.	G	G	C	AA	GA	G.
aro	T	C	G	C	-	T	A	T	A	-	A	C	T	-	-	-

***MYB3*-cds (1053 bp alignment)**

Group	947—	979—	1051—
Or	(CAC)x8+(CAG)x2	-	STOP (TGA)
On	-(CAG)x3	-	TGA
aus	-(CAG)x3	-	TGA
ind	(CAC)x6+CAG	STOP (TAG)	
jap	(CAC)x6+CAG	TAG	
tro	(CAC)x6+CAG	TAG	
aro	(CAC)x3+(CAG)x4	-	TGA

12 *GL3.2* (Os03g30420.1)

***GL3.2*-5′ (1157 bp alignment)**

Group	4	9	19	40	41	172	179	225	385	392	478	546	706	862
Or	C	G	G	G	T.	A	-	T	T	-	C	-	C	T
On	C	G	G	T	T.	A	-	T	T	-	C	-	C	T
aus	C	A	A	T	T.	A	A	T	C	-	C	-	T	A
ind	C/T	A/G	G	-/T	T./-	A/T	A	T	T	-/C.	C/-	-	C	T
jap	T	G	G	T	T.	A	A	T	T	C.	-	-	C	T
tro	T	G	G	-/T	T./-	T	A	T	T	C.	-	-	C	T
aro	C	G	G	G	T.	T	A	A	T	-	C	T.	C	T
							*							

***GL3.2*-cds (1554 bp alignment)**

Group	49	90	384	1480
Or	C.	C	C	G
On	C.	C	C	G
aus	-	C	C	G
ind	C.	T/C	T/C	C
jap	C.	T	T	C
tro	C.	T	T	C
aro	C.	C	C	G

13 *TAC3* (Os03g51660.1)

***TAC3*-5′ (1024 bp alignment)**

Group	181	187
Or	T	A
On	T	A
aus	-	A
ind	-/T	A
jap	T	T
tro	T	T
aro	-	A

***TAC3*-cds (459 bp alignment)**

Group	
Or	
On	
aus	
ind	identical in all sequences
jap	
tro	
aro	

14 *Hd6* (Os03g55389.1)

***Hd6*-5′ (2280 bp alignment)**

Group	12	91	197	271-	365	393	953	991	1093	1099	1133	1163	1169	1348
Or	C	-	-	-	-	-	-	-	-	-	-	-	-	-
On	C	C.	-	-	G	C	A	C	G	C	A	G	G	A
aus	C	C.	-	-	G	C	A	C	G	C	A	G	G	A
ind	C/T	C./C..	-/C.	-/A.	G/A	C/A	G	C/T	G/A	C/T	A/T	G/T	G/A	A/C
jap	T	C..	C.	A.	A	A	G	T	A	T	T	T	A	C
tro	T	C..	C.	A.	A	A	G	T	A	T	T	T	A	C
aro	T	C..	C.	A.	A	A	G	T	A	T	T	T	A	C

***Hd6*-cds (627 bp alignment)**

Group	436		625—627	
Or	A		TAA	STOP
On	A		TAA	STOP
aus	A		TAA	STOP
Ind	A		TAA	STOP
Jap	T	STOP		
tro	A		TAA	STOP
aro	A		TAA	STOP

15 *DST* (Os03g57240.1)

***DST*-5′ (1031 bp alignment, missing sites not counted)**

Group	86	91	266	391	479	490
Or	G	C	A	T	A	T
On	G	C	A	T	A	T
aus	G	C	G	A	T	T
ind	G	C	A	T	A	T
jap	T	T	A	T	A	-
tro	T	T	A	T	A	-
aro	T	T	A	T	A	-

***DST*-cds (928 bp alignment)**

Group	72	340	375	621
Or	-	G	C.	C.
On	-	G	C.	C.
aus	G	G	C.	C.
ind	-	G	C.	C.
jap	-	A	-	-
tro	-	A	-	-
aro	-	A	-	-

16 *CHI* (Os03g60509.1)

***CHI*-5′ (1400 bp alignment)**

Group	58	77	213	225	425	435	470-	544	489	1325	1328	1333
Or	A	A	C	T	T	A	AC	T	C	A	A	A
On	A	A	C	T	T	C	AC	T	C	A	A	A
aus	A	A	C	T	T	C	AC	T	C	A	A	A
ind	A	T	T	C	C	C	AC	T	T	C	A	T
jap	G	A	C	C	C	T	--	C	C	A	G	T
tro	G	A	C	C	C	T	--	C	C	A	G	T
aro	G	A	C	C	C	T	--	C	C	A	G	T

***CHI*-cds (762 bp alignment)**

Group	76	694	700-	703	760-	762
Or	A	T	TGA	STOP		
On	A	T	TGA	STOP		
aus	A	A..				STOP
ind	G	A..				STOP
jap	G	T	TGA	STOP		
tro	G	T	TGA	STOP		
aro	G	T	TGA	STOP		

1

d. Chromosome 4
(59 *trans* mutations)

17 ***GIF1*** (Os04g33740.1)

GIF1-5′(1238 bp alignment)

Group	235	367	869-
Or	..G.C..	G	(TA)x10
On	..G.C..	G	(TA)x15
aus	..G.C..	A	(TA)x14
ind	..A.T../-	G	(TA)x45,x44
jap	-	G	(TA)x28
tro	-	G	(TA)x22
aro	..G.C..	A	(TA)x14

GIF1-cds (1794 bp alignment)

Group	300	527	1173	1367
Or	G	G	A	T
On	G	G	A	T
aus	G	T	A	G
ind	G/A	G	A/G	T/G
jap	A	G	G	G
tro	A	G	G	G
aro	G	T	A	G
				*

18 ***Myb4*** (Os04g43680.1)

Myb4-5′(1008 bp alignment)

Group	26	216	231
Or	A	C	AGTTT
On	A	C	AGTTT
aus	C	T	-
ind	C	T	-
jap	C	T	-
tro	C	T	-
aro	C	T	-
	*	*	*

Myb4-cds (789 bp alignment)

Group	427	544—555	558
Or	GCC	TCGGCGTCCGCG	C
On	GCC	TCGGCGTCCGCG	C
aus	-	-	G
ind	-	-	G
jap	-	-	G
tro	-	-	G
aro	-	-	G
	*	*	*

19 ***An-2*** (Os04g43840.1)

An2-5′ (1000 bp alignment)

Group	84	380	766	873
Or	G	A	A	A
On	G	A	A	A
aus	A	G	T	C
ind	A	G	T	C
jap	A	G	T	C
tro	A	G	T	C
aro	A	G	T	C
	*	*	*	*

An2-cds (753 bp alignment)

Group	70	169
Or	C	G
On	C	G
aus	C	C
ind	-	G
jap	C	G
tro	-	C
aro	-	C

20 *Unknown* (Os04g47040)

Unknown-5′ (1010 bp alignment)

Group	122	126	621	873
Or	G	G	-	C
On	G	G	-	C
aus	T	A	G.	C
ind	G	G	-	C
jap	G	G	-	T
tro	G	G	-	T
aro	G	G	-	T

Unknown-cds (1695 bp alignment)

Group	565-	594	847-
Or	A..	C	-
On	A..	C	-
aus	-	C	-
ind	-	C	-
jap	-	G	AT..
tro	-	G	AT..
aro	-	G	AT..
	*		

21 ***AGO2*** (Os04g52540.1)

AGO2-5′ (1152 bp alignment)

Group	56	133-	224-	288	470-	522
Or	AA	C	-	-	T..	A
On	-	T	-	C	T..	A
aus	-	C	-	C	T..	A
ind	A-	C	T..	C	T..	A
jap	A-	--	T..	T	-	T
tro	A-	--	T..	T	-	T
aro	A-	--	T..	T	-	T

AGO2-cds (3124 bp alignment, partial presentation)

Group	483	632	1683	1817	1819	1840	1848—
Or	T	A	TG	T	GC	C	T
On	G	A	TG	G	GC	C	T
aus	G	C	TG	T	GC	C	T
ind	T	C	TG	T	GC	C	T
jap	A	C	CC	C	AG	G	A
tro	A	C	CC	C	AG	G	A
aro	T	A	TG	T	GC	C	T
		*					

22 ***IPK1*** (Os04g56580.1)

IPK1-5′(1000 bp alignment)

Group	37	316	382
Or	C	C	C
On	C	C	C
aus	C	C	C
ind	C	C	C
jap	T	G	C
tro	T	G	C
aro	T	G	T

IPK1-cds (1338 bp alignment)

Group	288
Or	C
On	C
aus	T
ind	T
jap	C
tro	C
aro	C

23 ***F3H***(Os04g56700.1)

F3H-5′ (1002 bp alignment)

Group	281	290	313	378	458	474
Or	A	-	G	G	A	C
On	A	-	G	G	A	C
aus	-	-	A	G	C	C
ind	-	-	A	G	C	C
jap	A	T	G	A	A	A
tro	A	T	G	A	A	A
aro	A	T	G	A	A	A

F3H-cds (1134 bp alignment)

Group	354	666	722	793—831	899	942
Or	C	C	G	T.	G	A
On	C	C	G	T.	G	A
aus	T	C	**G**	-	**G**	A
ind	T	C	**G**	T.	**G**	A
jap	C	**T**	C	T.	**T**	G
tro	C	**T**	C	T.	**T**	**G**
aro	C	**T**	C	T.	**T**	**G**

24 ***SH4*** (Os04g57530.1)

SH4- 5′ (1006 bp alignment)

Group	46	55	99	122	247	661	663
Or	C	A	G	G	C	G	A
On	C	A	G	G	C	G	A
aus	T	-	A	C	A	G	-
i-b	T	-	A	C	A	G	-
J-a	T	-	A	C	A	-	-
tro	T	-	A	C	A	-	A
aro	T	-	A	C	A	G	-
	*	*	*	*	*		

SH4-cds (1173 bp alignment)

Group	237
Or	G
On	G
aus	T
i-b	T
J-a	T
tro	T
aro	T
	*

e. Chromosome 5
(31 *trans* mutations)

25 ***GS5*** (Os05g06660.1)

GS5-5′ (1010 bp alignment)

Group	114	137	157	158
Or	T	G	-	-
On	G	G	-	-
aus	G	G	-	-
ind	G/A	G	T	T
jap	G	A	T	-
tro	G	A	T	-
aro	G	A	T	T

GS5-cds (1458 bp alignment, partial presentation)

Group	97-	137	386	1138	1204
Or	-	G	A.	G	A.
On	-	G	A.	G	A.
aus	-	G	A.	Missing	
ind	-	G	C.	G	-
jap	G	A	A.	G	-
tro	G	A	A.	G	-
aro	G	A	A.	G	-

26 ***ACS3*** (Os05g10780.1)

ACS3-5′ (1000 bp alignment)

Group	35	38	130	330	738	901	953
Or	T	AG	G	C	C	G	C
On	T	AG	G	C	C	G	C
aus	T	AG	G	C	C	G	C
ind	C	AG	G	C	C	A	T
jap	T/C	TA/AG	A/G	C	T/C	A/G	T/C
tro	T	AG	A	A	C	G	C
aro	T	TA	A	C	T	G	C

ACS3-cds (1314 bp alignment)

Group	105	196	258	731	829	1188	1297
Or	C	T	G	G	G	C	G
On	C	T	G	G	G	C	G
aus	C	T	A	G	G	C	G
ind	C	G	G	A	A	C	G
jap	A/C	G/T	G	G/A	G/A	C	G
tro	C	T	G	G	G	T	T
aro	A	G	G	G	G	C	G

27 ***SH5*** (Os05g38120.1)

SH5-5′ (1466 bp alignment)

Group	6	176	265	300	788	1205
Or	C	T	G	T	-	-
On	C	T	G	T	-	-
aus	C	T	G	T	GTA	-
ind	C	C	G	A	-	-
jap	T	T	T	T	-	A..
tro	T	T	T	T	-	A..
aro	T	T	T	T	-	A..

SH5-cds (1743 bp alignment)

Group	72	277	427	934	1038	1341
Or	C	G	GGC	G	T	A
On	-	G	GGC	G	T	A
aus	-	G	GGC	G	T	A
ind	C	T	GGC	G	T	A
jap	G	G	GGC	A	A	G
tro	G	G	GGC	G	A	G
aro	G	G	-	G	A	G

28 ***TPS1***(Os05g44210.1)

TPS1-5′ (1018 bp alignment)

Group	3	87	91	104	177	348	416	432	492	722
Or	G	-	G	G	-	A	-	C	A	2A
On	G	-	G	G	-	A	-	C	A	2A
aus	G	-	G	A	-	T	-	C	A	1A
ind	G	-	A	G	-	A	T..	C	A	0A
jap	A	T	G	G	T	A	-	T	C	3A
tro	A	T	G	G	T	A	-	T	C	2A
aro	A	T	G	G	T	A	-	T	C	2A

TPS1-cds (2595 bp alignment)

Group	40-42	88-99	1164	1584	1662
Or	TTC	T..	T	T	C
On	TTC	T..	T	T	C
aus	TTC	T..	T	T	C
ind	TTC	T..	T	G	C
jap	-	-	A	T	T
tro	-	-	A	T	T
aro	-	-	A	T	T

f. Chromosome 6
(24 *trans* mutations)

29 ***EPSPS***(Os06g04280.1)

EPSPS-5′ (1007 bp alignment)

Group	12	38	294
Or	G	C	T
On	G	C	T
aus	G	C	T
ind	C/G	G/C	T/-
jap	C	G	-
tro	C	G	-
aro	C	G	-

EPSPS-cds (1548 bp alignment)

Group	162
Or	G
On	G
Aus	T
ind	T/G
jap	G
tro	G
aro	G

30 ***Hd3a***(Os06g06320.1)

Hd3a-5′ (1023 bp alignment)

Group	317	336	482—	513	599	728—	862	942	995
Or	T	C	G..	T	G	-	G	C	T
On	T	C	G..	T	G	GA	G	C	T
aus	C	C	-	T	A	-	G	C	A
ind	C	C	-	T	A	-	G	C	A
jap	C	C	-	T	A	-	G	C	A
tro	T	A	G..	C	G	GAGA	G	A	T
aro	T	A	G..	C	G	GA	T	A	T

Hd3a-cds (540 bp alignment)

Group	510	535-536
Or	C	AA
On	C	AA
aus	C	AA
ind	C	AA
jap	A	CC
tro	A	CC
aro	C	CC

31 ***C1***(Os06g10340.1)

C1-5′ (1002 bp alignment)

Group	397	990—
Or	C	(AG)x4
On	C	-
aus	T	(AG)x3
ind	T	(AG)x3
jap	T	(AG)x3
tro	T	(AG)x3
aro	T	(AG)x3
	*	*

C1-cds (819 bp alignment)

Group	122	208	679—692	789
Or	C	A.	-	-
On	C	A.	T.	C.
aus	G	A.	T.	C.
ind	C	A.	T.	C.
jap	C	A.	T.	C.
tro	C	-	T.	C.
aro	C	A.	T.	C.

32 ***TCP19***(Os06g12230.1)

TCP19-5′ (1116 bp alignment)

Group	27	48	70	320	453	463	502	518	527—	530—	532—	623—	657	742	773—	849	946—	1089
Or	-	C	T	G	G	-	G	C	T.	CT	-	G.	T	-	-	T	G.	-
On	-	C	T	G	G	-	G	C	T.	CT	-	G.	T	-	-	T	G.	-
aus	A	C	-	A	G	-	G	C	T.	-	-	G.	A	-	-	T	G.	-
ind	-	T	-	G	G	-	G	C	T.	CT	-	G.	T	-	-	T	G.	-
jap	-	T	-	G	G	-	G	C	T.	CT	CT	G.	T	-	-	T	G.	-
tro	-	T	-	G	G	-	G	C	T.	CT	CT	G.	T	-	-	T	G.	-
aro			missing		A	A	A	T	-	-	-	-	T	A	AG	C	-	GT
			*															

TCP19-cds (1209 bp alignment)

Group	35	125—129	132	157—192	216	448	558	819	903	912	1014	1147	1166
Or	A	-	C	-	G	T	C	G	C	A	C	C	G
On	G	-	C	G.	G	T	C	G	C	A	C	C	G
aus	G	-	C	G.	T	G	C	G	T	T	C	T	G
ind	A	-	C	-	G	T	C	G	C	A	C	C	G
jap	A	-	C	-	G	T	C	G	C	A	C	C	G
tro	A	-	C	-	G	T	C	G	C	A	C	C	G
aro	G	G.	T	G.	G	T	T	C	C	A	T	C	T

33 ***Hd1***(Os06g16370.1)

Hd1-5′ (1378 bp alignment)

Group	216	421	476	648	1103	1150	1172—
Or	C	C	G	C	C	C	C.
On	C	C	G	C	C	C	C.
aus	A	C	G	A	C	G	-
ind	C	T	G	C	A	C	C.
jap	C	C	G	C	A	C	C.
tro	C	C	A	C	C	C	C.
aro	C	C	A	C	C	C	C.

Hd1-cds (1347 bp alignment)

Group	466	487	511—627	628—666	994	1185—1351
Or	C	A	AG.	A.C.	TT	T.G.
On	C	A	AA.	A.T.	TT	T.A.
aus	A	A	-	-	-	STOP
ind	C	G	A.	A.C./A.T.	TT	T.G.
jap	C	G	A.	A.T.	TT	T.G.
tro	A	A	-/G.	A.C./-	-/TT	STOP/T.G.
aro	A	A	-	-	TT	T.G.

One mutation

1

g. Chromosome 7
(26 *trans* mutations)

34 ***PROG1***(Os07g05900.1)

PROG1-5′ (1031 bp alignment)

Group	168	821
Or	-	-
On	-	T.
aus	-	A.
ind	T	A.
jap	T/-	A.
tro	-	A.
aro	-	A.
		*

PROG1-cds (514 bp alignment)

Group	99
Or	A
On	A
aus	G
ind	G
jap	G
tro	G
aro	G
	*

35 ***Rc***(Os07g11030.1)

Rc-5′ (594 bp alignment)

Group	7	207	362	363	450
Or	T	C	T	T	G
On	T	C	T	T	G
aus	T	C	-	-	G
ind	T	C/A	-/T	-	G/T
jap	T	A	T	-	T
tro	-	A	T	-	T
aro	-	A	T	-	T
				*	

Rc-cds (2013 bp alignment)

Group	21	96	1408—1421	1437	1833—1844	1962
Or	A	G	AC..	T	-	C
On	A	G	AC..	T	(CGG)x2	C
aus	A	T	AC..	T	(CGG)x4	C
ind	A/T	G	-	T	-	T
jap	T	G	-	-/T	-	T
tro	T	G	-	T	-	T
aro	T	G	-	T	-	T

36 ***SPL13***(Os07g0505200)

SPL13-5′ (1014 bp alignment)

Group	112	122	459	478	516	827	837
Or	T	G	C	C	G	C	T..
On	T	G	C	G	G	C	T..
aus	T	G	G	G	G	C	T..
ind	-/T	G	C/G	G	G	C	T..
jap	T	G	C	A	G	C	-
tro	-	A	C	G	A	-	T..
aro	T	G	G	G	G	C	T..

SPL13-cds (651bp alignment)

Group	441	593	612
Or	C	A	C
On	C	A	C
aus	C	A	T
ind	C	A	C
jap	T	A	C
tro	C	G	T
aro	C	A	T

37 ***WG7***(Os07g47360.1)

WG7-5′ (1064 bp alignment)

Group	65	80	108	334	345	498	521-	794
Or	C	A	-	A	C	C	A	A
On	C	A	-	A	C	C	A	A
aus	C	A	-	A	C	C	A	A
ind	C/T	A/G	-/T	A/T	C/T	C/T	A/-	A/-
jap	T	G	-/T	T	T	T	-	-
tro	T	G	T	T	T	T	-	-
aro	T	G	T	T	T	T	-	-

WG7-cds (4803 bp alignment)

Group	512	637	2002	2126
Or	C	G	G	T
On	C	G	G	A
aus	C	G	G	A
ind	C/T	G/A	G/A	A/G
jap	T	G/A	A	G
tro	T	A	A	G
aro	T	A	A	G

h. Chromosome 8
(11 *trans* mutations)

38 ***RAE2***(Os08g37890.1)

RAE2-5' (1003 bp alignment)

Group	237	304	993—995
Or	C	T	GCT
On	C	T	GCT
aus	T	G	-
ind	T	G	-
jap	T	G	-
tro	T	G	-
aro	T	G	-
	*	*	*

RAE2-cds (593 bp alignment)

Group	297	303	304—305	353—355	395—397	408—410	591—593
Or	C	C	GC	T	stop		
On	C	C	GC	T	stop		
aus	-	-	-	STOP			
ind	C	C	-	T		STOP/-	STOP/
jap	-/C	-/C	-	-/STOP			STOP/
tro	C	C	-	T			STOP
aro	-	-	-	STOP			
			*				

39 ***SPL16***(Os08g41940.1)

SPL16-5' (1017 bp alignment)

Group	317	714	737	746	979—988
Or	C	C	T	G	C.
On	C	C	T	G	C.
aus	C	C	T	G	C.
ind	T	T/C	-	G/-	C.
jap	C	T	-	-	-
tro	C	T	-	-	-
aro	C	T	-	-	-

SPL16-cds (1368 bp alignment)

Group	36	329—331	821
Or	C	-	C
On	C	-	C
aus	C	-	C
ind	T	-	A
jap	C	CGG	C
tro	C	CGG	C
aro	C	CGG	C

i. Chromosome 9

(21 *trans* mutations)

40 *Unkown* (Os09g26890.1)

***09g26890*-5′ (1075 bp alignment)**

Group	341	417	485	647	744	849	857	1045—1047	1063
Or	G	-	G	G	G	C	C	T.	-
On	G	-	G	G	G	C	C	T.	-
aus	G	-	G	G	G	C	C	T.	-
ind	G	-	A	G	A	C	T	T.	-
jap	G	-	G	A	G	T	C	-	-
tro	A	C	G	A	G	T	C	-	C
aro	A	-	G	A	G	T	C	-	-

***09g26890*-cds (360 bp alignment)**

Group	21	34	81	242	250—263
Or	C	G	C	C	-
On	C	G	C	C	-
aus	C	G	C	C	-
ind	T	A	C	C	G.
jap	C	G	T	T	-
tro	C	G	T	T	-
aro	C	G	T	T	-

41 ***DEP1***(Os09g26999.1)

***DEP1*- 5′ (1000 bp alignment)**

Group	94	267	498	508
Or	G	G	C	C
On	G	G	C	C
aus	G	G	C	C
ind	G	G	C	C
jap	T	-	A	G
tro	T	-	A	G
aro	T	-	A	G

***DEP1*-cds (1281 bp alignment)**

Group	611	833
Or	G	C
On	G	C
aus	A	G
ind	G/A	C/G
jap	G	C
tro	G	C
aro	G	C

42 ***PGI***(Os09g29070.1)

***PGI*-5′ (1000 bp alignment)**

Group	330	750	814	993
Or	C	G	T	C
On	C	G	T	C
aus	C	G	T	C
ind	C	A	T	T
jap	A	G	C	C
tro	A	G	C	C
aro	A	G	C	C

***PGI*-cds (1878-bp alignment)**

Group	201	321	1693	1783
Or	G	G	T	G.
On	G	G	T	G.
aus	G	G	C	-
ind	T	C	T	G.
jap	G	G	T	G.
tro	G	G	T	G.
aro	G	G	T	G.

43 ***PRR95*** (Os09g36220.1)

***PRR95*-5′ (1034 bp alignment)**

Group	67	184	283	287	289	294	297	301	886	930
Or	C	A	G	A	C	C	-	A	A	GA
On	C	A	G	A	C	C	C	A	A	GA
aus	A	A	A	A	G	C	A	A	A	-
ind	C	C	G	C	C	C	C	-	T	-
jap	C	A	G	A	C	-	-	A	A	-
tro	C	A	G	A	C	-	-	A	A	-
aro	C	A	G	A	C	-	-	A	A	-
										*

***PRR95*-cds (1872 bp alignment)**

Group	9	407	999
Or	A	G	C
On	A	G	C
aus	A	G	C
ind	T	G	C
jap	T	G	T
tro	T	A	T
aro	T	A	T

44 ***DHQS*** (Os09g36800.1)

***DHQS*-5′ (1013 bp alignment)**

Group	956
Or	A
On	A
aus	A
ind	A
jap	C
tro	C
aro	C

***DHQS*-cds (1332 bp alignment)**

Group	1014
Or	C
On	C
aus	C
ind	C
jap	T/C
tro	C
aro	C

1

j. Chromosome 10

(9 *trans* mutations)

45 ***DAHPS2*** (Os10g41480.1)

DAHPS2-5’ (1007 bp alignment)

Group	104	141—145	155	680	990
Or	C	C.	T	C	G
On	C	C.	T	C	G
aus	-	-	C	T	A
ind	-	-	C	T	A
jap	-	-	C	T	A
tro	-	-	C	T	A
aro	-	-	C	T	A
	*	*	*	*	*

DAHPS2-cds (1509 bp alignment)

Group	174
Or	G
On	G
aus	A
ind	A
jap	A
tro	A
aro	A
	*

46 ***MYC2***(Os10g42430.1)

MYC2-5’ (1015 bp alignment)

Group	208..	221	588
Or	T..	T	A
On	T..	T	A
aus	T..	T	G
ind	T..	T	G
jap	-	G	A
tro	-/T..	G/T	A
aro	-	G	A

MYC2-cds (2256 bp alignment)

Group	272
Or	-
On	-
aus	-
ind	-
jap	-
tro	-
aro	CGC

k. Chromosome 11
(20 *trans* mutations)

47 ***PK1***(Os11g05110.2)

PK1-5′ (1016 bp alignment)

Group	104	284	342	394	405	427	455	622	629	809	932—934
Or	A	C	G	C	T	C	G	C	A	C	-
On	A	C	G	C	A	C	G	C	-	C	-
aus	A	C	G	C	A	C	G	C	-	C	-
ind	A	C	G	C	-	A	G	G	-	C	AAG
jap	A	T	A	-	-	T	A	C	G	T	AAG
tro	A	T	A	A	-	T	A	C	G	T	AAG
aro	-	C	G	C	-	A	G	G	-	C	AAG

PK1-cds (1584 bp alignment) gene of 15 introns

Group	261	1269
Or	T	A
On	C	C
aus	C	C
ind	C	A
jap	T	A
tro	T	A
aro	C	A

48 ***Unknown***(Os11g07910.1)

Unknown-5′ (1004 bp alignment)

Group	177	255	313—320	371	525	579—588	665	674	879
Or	-	G	A.	G	T	T.	C	C	C
On	-	G	A.	G	-	T.	C	C	C
aus	-	G	A.	A	-	T.	C	C	C
ind	-	G	A.	G	-	T.	C	C	C
jap	T	A	-	G	C	-	T	T	-
tro	T	A	-	G	C	-	T	T	-
aro	-	G	A.	G	-	T.	C	C	C

Unknown-cds (1782 bp alignment)

Group	
Or	
On	
aus	
ind	No mutations
jap	
tro	
aro	

49 ***CHS*** (Os11g32650.1)

CHS-5′ (1254 bp alignment)

Group	102	138	218	251	281	585	732—785	799	894
Or	G	C	G	C	G	T	A.	G	G
On	G	C	G	C	G	T	A.	G	G
aus	T	T	A	T	T	A	A.	A	A
ind	G	C	A/G	C	G	T	A.	G	G
jap	G	C	G	C	G	T	-	G	G
tro	G	C	G	C	G	T	-	G	G
aro	G	C	G	C	G	T	-	G	G

CHS-cds (1197 bp alignment)

Group	6	18	57	1179
Or	G	G	G	C
On	G	G	G	C
aus	A	A	A	T
ind	A/G	G	A/G	C
jap	G	G	G	C
tro	G	G	G	C
aro	G	G	G	C

I. Chromosome 12
(5 *trans* mutations)

50 ***Pi-ta*** (Os12g18360.1)

Pi-ta-5′ (1277 bp alignment)

Group	240	282	289	338	371	892	900
Or	A	T	T	C	-	G	G
On	A	T	T	C	-	G	G
aus	A	T	T	C	-	G	G
ind	A	T	T	C	-	G	T
jap	A	C	-	C	-	A	G
tro	T	T	-	T	G.	G	G
aro	A	T	T	C	-	G	G

Pi-ta-cds (2787 bp alignment)

Group	444	474	527	2388
Or	G	C	C	G
On	G	C	A	G
aus	G	C	A	G
ind	G	C	A	G
jap	C	G	T	A
tro	G	C	A	G
aro	G	C	A	G

51 ***Unknown***(Os12g34860.1)

Unknown-5′ (1013 bp alignment)

Group	981—989	998—999
Or	-	-
On	-	-
aus	-	-
ind	-/C	AA
jap	C	AA
tro	C	AA
aro	C	AA

unknown-cds (1348 bp alignment)

Group	1078	1080	1120	1348
Or	G	A	STOP	
On	G	A	STOP	
aus	-	T	A.	STOP
ind	-	T	A.	STOP
jap	-	T	A.	STOP
tro	-	T	A.	STOP
aro	-	T	A.	STOP
	*	*		

Figure S1. Identification of rice mutations across 51 sampled genes. The identity of a mutation was assigned by its presence in at least one subgroup but absence in both genomes of parental species including *O. rufipogon* (Or) and *O. nivara* (On). A mutation can be an insertion or deletion of any size in continuity, or substitution (a nucleotide followed by "." means more than one base pair (bp) in comparison). Adjacent changes of indel or substitution were considered from one mutational event and an indel causing a shift of stop codon as one event. These occurred in two subgroups are marked in green, in three subgroups (including *aromatic* (aro)) in orange or blue (other cases). When the same mutations present in at least three subgroups include *indica* (*sensu stricto*, ind) and *japonica* (*sensu stricto*, jap) but not *aromatic*, they are marked in light purple; when five subgroups have the same mutations, they are marked in dark purple and asterisks. Mutations that occurred in only one subgroup are marked in grey. Transient mutations in *indica* (*sensu stricto*), if not also present in another subgroup, were generally not presented. **a** — **l** chromosome 1 — chromosome 12.

a

Sources	Location of markers (bp)					
	466[b]	994,995	1185	1231	1340	1345
aus	A	--	STOP	C	A	T
***tropical japonica* (CM)**[a]	A	TT	T	A (STOP)	G	T
aromatic	A	TT	T	C	G	STOP

[a] The locus is polymorphic in *tropical japonica*, and CM here refers to the type found in varieties such as Chao Meo.
[b] The site in *indica* (*sensu stricto*) and *japonica* (*sensu stricto*) is C.

b

```
                    10        20        30        40        50        60        70        80        90
            ---------+---------+---------+---------+---------+---------+---------+---------+---------+
aus          MNYNFGGNVFDQEVGVGGEGGGGGEGSGCPWARPCDGCRAAPSVVYCRADAAYLCASCDARVHAANRVASRHERVRVCEACEQAPAALAC   268
tropical-K   MNYNFGGNVFDQEVGVGGEGGGGGEGSGCPWARPCDGCRAAPSVVYCRADAAYLCASCDARVHAANRVASRHERVRVCEACEQAPAALAC   268
tropical-CM  MNYNFGGNVFDQEVGVGGEGGGGGEGSGCPWARPCDGCRAAPSVVYCRADAAYLCASCDARVHAANRVASRHERVRVCEACEQAPAALAC   268
aromatic     MNYNFGGNVFDQEVGVGGEGGGGGEGSGCPWARPCDGCRAAPSVVYCRADAAYLCASCDARVHAANRVASRHERVRVCEACEQAPAALAC   268

                    100       110       120       130       140       150       160       170       180
            ---------+---------+---------+---------+---------+---------+---------+---------+---------+
aus          RADAAALCVACDVQVHSANPLARRHQRVPVAPLPAITIPATSVLAEAVVATATVLGGKDEEVDSWIILSKDSNNNNNNNNSNSSNN----   526
tropical K   RADAAALCVACDVQVHSANPLARRHQRVPVAPLPAITIPATSVLAEAVVATATVLGGKDEEVDSWIILSKDSNNNNNNNNSNSSNNGMYF   538
tropical CM  RADAAALCVACDVQVHSANPLARRHQRVPVAPLPAITIPATSVLAEAVVATATVLGGKDEEVDSWIILSKDSNNNNNNNNSNSSNN----   526
aromatic     RADAAALCVACDVQVHSANPLARRHQRVPVAPLPAITIPATSVLAEAVVATATVLGGKDEEVDSWIILSKDSNNNNNNNNSNSSNN----   526

                    190       200       210       220       230       240       250       260       270
            ---------+---------+---------+---------+---------+---------+---------+---------+---------+
aus          ------------------------------------------------GMYFGEVDEYFDLVGYNSYYDNRIENNQDQQYGMHEQQEQQQ   652
tropical K   GEVDEYFDLVRYNSYYDNNNNDNSNSNSSNNDNDNDNDNNNNSNSSNNGMYFGEVDEYFDLVGYNSYYDNRIENNQDQQYGMHEQQEQQQ   808
tropical CM  ------------------------------------------------GMYFGEVDEYFDLVGYNSYYDNRIENNQDQQYGMHEQQEQQQ   652
aromatic     ------------------------------------------------GMYFGEVDEYFDLVGYNSYYDNRIENNQDQQYGMHEQQEQQQ   652

                    280       290       300       310       320       330       340       350       360
            ---------+---------+---------+---------+---------+---------+---------+---------+---------+
aus          QQQEMQKEFAEKEGSECVVPSQITMLSEQQHSGYGVVGADQAASMTAGVSAYTDSISNSISLINGGGYSTRQHGDRYAKFQHPDTCWSNQ   922
tropical K   QQQEMQKEFAEKEGSECVVPSQITMLSEQQHSGYGVVGADQAASMTAGVSAYTDSISNSISLINGGGYSTRQHGDRYAKFQHPDTCWSNQ   1078
tropical CM  QQQEMQKEFAEKEGSECVVPSQITMLSEQQHSGYGVVGADQAASMTAGVSAYTDSISNSISFSSMEAGIVPDSTVIDMPNSSILTPAGAI   922
aromatic     QQQEMQKEFAEKEGSECVVPSQITMLSEQQHSGYGVVGADQAASMTAGVSAYTDSISNSISFSSMEAGIVPDSTVIDMPNSSILTPAGAI   922

                    370       380       390       400       410       420       430       440
            ---------+---------+---------+---------+---------+---------+---------+---------+---------
aus          SLLRSLASDVPSLQLHGQGGQGAQVQGEEEGQEV.                                                          1027
tropical K   SLLRSLASDVPSLQLHGQGGQGAQVQGEEEGQEV.                                                          1183
tropical CM  NLFSGPSLQMSLHFSSMDREARVLRYREKKKARKFEKTIRYETRKAYAEA.                                          1075
aromatic     NLFSGPSLQMSLHFSSMDREARVLRYREKKKARKFEKTIRYETRKAYAEARPRIKGRFAKRSDVQIEVDQMFSTAALSDSSYGTVPWF.   1189
```

Figure S2. The coding regions of *OsHd1* compared among three subgroups. **a** Alignment of the coding sequences of *OsHd1*. More details see Fig. S1. Two events of recombination (by arrow) between *aus* and *tropical japonica* were identified, which reconnected parental segments (high-lighted in orange) of the gene to give rise to the allele of *aromatic*. **b** Alignment of OsHd1 proteins. The alignment was conducted via Clustal W. Two sequences of *tropical japonica* (*tropical*) were from varieties Ketan (K) and Chao Meo (CM), and the type of CM served as one of the parental donors to *aromatic*.

a

***BADH2*-5'**

Group	5	32	45	191	256	278	355	363	365	382	475	508	536	647	673	675	685	765	879	944
Or	C	A	G	C	G	G	C	C	C	G	G	T	C	G	-	A	A	T	C	C
On	C	A	G	C	G	G	C	C	C	G	G	T	C	G	-	T	A	T	C	C
aus	T	G	C	C	A	A	T	C	C	A	A	T	T	G	TT	G	G	A	A	C
ind	C	A	G	C	G	G	C	C	T	G	G	C	C	G	-	T	A	T	C	C
jap	C	A	G	C	G	G	C	T	C	G	G	T	C	G	-	A	A	T	C	C
tro	C	A	G	T	G	G	C	C	C	G	G	T	C	A	-	T	A	T	C	-
aro	C	A	G	C	G	G	C	C	C	G	G	T	C	-	-	A	A	T	C	C

b

***BADH2*-cds**

Group	111—252
Or	C.
On	C.
aus	-
ind	C.
jap	C.
tro	C.
aro	C.

Figure S3. Mutation distributions at the locus of *BADH2* (Os08g32870.1). **a** The 5' region of *BADH2* in an alignment of 1052 bp. The sites are numbered from 5' to 3' upstream of the translation starting site (not including ATG). Subgroup-specific mutations are marked in grey. **b** The coding regions of *BADH2* in an alignment of 1512 bp. The deletion (-) is indicated by its position in the coding sequence starting with 1 at the first nucleotide of ATG. The format of group names follows Fig. 1 and Fig. 3b.
